# Self-Healing Oxalamide Organogelators of Vegetable Oil

**DOI:** 10.3390/gels9090699

**Published:** 2023-08-29

**Authors:** Nataša Šijaković Vujičić, Josipa Suć Sajko, Lidija Brkljačić, Petra Radošević, Ivanka Jerić, Ivona Kurečić

**Affiliations:** 1 Division of Organic Chemistry, Ruđer Bošković Institute, Bijenička 54, HR-10000 Zagreb, Croatia; pmance@irb.hr; 2 Laboratory for Biomimetic Chemistry, Ruđer Bošković Institute, Bijenička 54, HR-10000 Zagreb, Croatia; josipa.suc@irb.hr (J.S.S.); lidija.brkljacic@irb.hr (L.B.); ijeric@irb.hr (I.J.); kurecic.i@gmail.com (I.K.)

**Keywords:** organogelators, oxalamide, oil, thixotropy, fat substitutes, controlled drug delivery

## Abstract

The aim of this study was to assess the gelling potential of chiral oxalamide derivatives in vegetable oils. Special emphasis was given to the potential applications of the examined oil gels as sustained delivery systems and as fat substitutes in food products. The applicability of oil gelators is envisaged in food, cosmetics, and the pharmaceutical industry. The regulations requiring the elimination of saturated fats and rising concerns among consumers health motivated us to investigate small organic molecules capable of efficiently transforming from liquid oil to a gel state. The oxalamide organogelators showed remarkable gelation efficiency in vegetable oils, thermal and mechanical stability, self-healing properties, and a long period of stability. The physical properties of the gels were analysed by TEM microscopy, DSC calorimetry, and oscillatory rheology. The controlled release properties of acetylsalicylic acid, ibuprofen, and hydrocortisone were analysed by the LC–MS method. The influence of the oil type (sunflower, soybean, and olive oil) on gelation efficiency of diverse oxalamide derivatives was examined by oscillatory rheology. The oxalamide gelators showed thermoreversible and thixotropic properties in vegetable oils with a minimum gelation concentration of just 0.025 wt%. The substitution of palm fats with gelled sunflower oil applied in cocoa and milk spreads at gelator concentrations lower than 0.2 wt% have shown promising viscoelastic properties compared to that of the original food products.

## 1. Introduction

Low molecular weight organic gelators (LMWOGs) represent a dynamic class of soft matter systems [1,2,3]. In the last three decades, hundreds of organogelators have been prepared and characterized as potential stimuli responsive materials [4,5]. Nonetheless, thixotropy has remained the least investigated property due to the low probability that organogelators possess that physical property. Low molecular weight organic gelators are molecules capable of forming unidirectional self-assembled aggregates, specifically through no-covalent interactions [6], intertwining in a three-dimensional network of fibres in different solvents and consequently preventing the flowing properties of the solvent used [7,8]. The driving forces of the self-assembly can include hydrogen bonding, π–π stacking, donor–acceptor interactions, and van der Waals interactions [9]. Different classes of LMWOGs have been reported, although the most common group is based on hydrogen bonding [10]. Gelation properties of chiral oxalamide derivatives have been extensively studied during the last twenty years by Žinić et al. [11]. The oxalamide molecules are connected by strong intermolecular hydrogen bonds between planar oxalamide units [12]. The influence of the hydrogen bonding unit, the number of those units in the molecule, the types of chiral centres, and the influence of lipophilic or hydrophilic terminal groups on gelation properties have been studied in detail. 

Thixotropy is a fascinating feature present in some organogel systems that has attracted a lot of attention [13]. Self-healing gels can disintegrate in a solution under an external mechanical stress and can regain their elastic properties upon removal of the stress [14]. Thixotropic materials are useful in the food industry and as bio- and self-healing materials [15]. Thixotropic hydrogels have been used in various therapeutic applications, including drug delivery [16]. Correlations between thixotropic and structural properties of molecular gels with crystalline networks [17], the remarkable role of hydrogen bonds, halogen, and the solvent effect on self-healing supramolecular gels [18], and an influence of H-bonding interactions on viscoelasticity and thixotropy of molecular gels have provided new insights toward the understanding of gelation mechanisms [19]. The study performed on the amide and amine LMWOGs based on (R)-12-hydroxystearic acid (12HSA) has indicated that the degree of viscoelastic recovery can be correlated qualitatively with the strength of hydrogen-bonding interactions among the gelator molecules [20]. Furthermore, the degree of viscoelastic recovery is not very sensitive to the amount of destructive strain applied to the gels, meaning the self-assembled fibre network is disrupted but not totally destroyed [17]. The H-bonding interactions between the gelator molecules and gelator–liquid interactions in the gel state and in the sol state influence the different stages of aggregation, nucleation, and growth of the aggregates forming the self-assembled fibrillar network. It appears that destructive strain breaks the intermolecular interactions near the junction zones of the self-assembled fibrillar network without disassembling the individual fibres. The thixotropic behaviour was observed due to the reformation of the connections between 1D objects after the cessation of shear, which means that 1D objects are retained in large part and H-bonding groups at their surface allow relatively indiscriminate reassembly into the 3D self-assembled fibrillar network [20]. Nonetheless, the development of self-healing LMWOGs is still a challenging task because of the lack of in-depth studies about self-healing mechanisms of gels and the solvent effect on gel properties. 

Thermal reversibility and sensitivity to external stimuli makes molecular gels attractive candidates for applications in the cosmetic [21], drug delivery [22], and food industries [23].

In the last two decades, the especially attractive gels became the so-called oleogels [24]. Driven by the need for trans and saturated fat replacement, the field of fat substitutes has made tremendous advances [25]. Saturated fats provide the structure and functionality for processed food products. However, the replacement of saturated fats by unsaturated oils in processed food products is not simple as liquid oil can negatively affect the texture of food products. An investigation has showed that replacement of saturated fats with poly unsaturated fatty acids has clear health benefits in the improvement of the nutritional profile of foods [26].

Fat mimetics, or structured oil materials, are designed to enhance the nutritional profile of lipid-based food products. The term fat mimetics includes the non-chemical transformation of liquid oil into the solid state, which could mimic the physical properties and functionality of solid fats. Fat mimetics include polymeric networks of ethylcellulose, emulsion-templated networks of proteins and polysaccharides, colloidal and self-assembled fibrillar networks of polar lipid crystals, and solid o/w emulsions of oil trapped within crystallized lamellar mesophases [27]. Research of new formulations includes bakery products, chocolate, dairy products, meat products, margarines, and spreads [28]. It seems that oleogels may be a promising solution for replacing or reducing saturated fats, since they have shown the potential to prevent oil leakage and influence the encapsulation and/or controlled release of hydrophobic bioactive molecules [29]. 

Oleogels are prepared by the addition of a structuring agent to liquid vegetable oil, followed by a heating–cooling procedure until a 3D network capable of entrapping liquid oil in a gel-like state is formed. The oleogel characteristics vary depending on parameters such as gelator structure, concentration, oil type, and preparation conditions [30]. The influence of the oil type regarding chemical composition, polarity, and viscosity offers new perspectives in the creation of diverse functional solutions [31]. The potential of using organogels as an alternative oil-structuring method has been investigated using different gelator–oil systems. 

In many oleogels, a gelation process is achieved by low molecular weight organogelators such as waxes [32], lecithin [33], phytosterols [34], monoglycerides [35], and ethylcellulose [36]. Attention was also paid to triacylglyceride derivatives [37], diacylglycerides, free fatty acids, n-alkanes, and fatty alcohols and their mixtures [38], due to their biocompatibility and ability to form a crystal network [39]. One of the most investigated systems was food-grade polymer oleogels [40]. Recently, the role of polar head groups and aliphatic tails of different low molecular weight molecules emphasized the involvement of hydrogen bonds and van der Waals interactions in the self-assembly process occurring in oil [41].

Research on fat mimetics as the potential replacement of saturated fats in food products is still in progress [28]. Despite certain promising functional properties of organogelators, their application in the food industry remains a challenge, including production costs, availability, and formal approval as additives by regulatory authorities.

However, the applicability of oil gelators possessing, amongst others, thixotropic properties can be foreseen not only in food industry but also in the cosmetics and pharmaceutical industries as sustained drug delivery systems [42]. There is a great need to discover functional oil gelators with potent gelation efficacy, a self-healing nature, and proper thermodynamic and rheological properties applicable in different areas.

In this study, we have examined the gelation properties of different chiral oxalamide derivatives in various oils (sunflower, soybean, and olive oil). Thixotropic properties of oxalamide derivatives were examined in different oils and the most potent gel systems were explored as sustained drug delivery vehicles. Finally, the oxalamide derivative was used as a fat substitute in the gelation of various food processed spreads to emphasise the potential of self-assembled nanostructures in mimicking the original products. 

## 2. Results and Discussion

### 2.1. Design, Gelation Properties, Thixotropy, and Stability

Oxalamides represent persistent gelation generating units due to conformational rigidity and strict in-plane intermolecular hydrogen bonding between the oxalamide units. The influence of stereochemistry on the gelation properties of bis (amino acid) oxalamide gelators is reflected through the gelation via self-assembly of optically pure gelators. The oxalamides exhibited a bilayer or inversed-bilayer type of organisation in the gelator assemblies formed in hydro and organogels, respectively [11]. Incorporation of two amino acid–oxalamide units into conformationally more flexible structures bridged by methylene bridges has endowed gelators capable of forming diverse aggregates of achiral and chiral morphologies [43], although the capacity of the gelator network of the bridged bis-oxalamides in various organic solvents was quite poor. Numerous oxalamide derivatives were subjected to gelation tests conducted in sunflower oil. The results of the gelation experiments of different chiral oxalamide derivatives in various oils were patent protected [44]. The bis (amino acid) oxalamide derivatives showed a lack of uniformity regarding gelation behaviour in sunflower oil (Table S1). One would expect to observe small differences in the gelation abilities of leucine and valine oxalamide derivatives. Besides, reasonable comparisons between certain acid, amide, or ester derivatives were expected. The conducted gelation experiments once again showed uncertainty in prediction in specific situations as is gelation in oil as a complex solvent. The bis (valine carboxylic acid) oxalamide derivative showed powerful gelation of sunflower oil, contrary to the absence of gelation property in the bis (leucine carboxylic acid) oxalamide derivative. The bis (leucine amide) derivative was a prominent gelator in sunflower oil, contrary to the poor gelation observed in the bis (valine amide) derivative (Table S1). Bis (phenylalanine amide) showed powerful gelation capacity but without thixotropic properties. The ester derivatives were generally assessed as poor or no gelators at all. The presented results showed that the highest possibility for the occurrence of gelation behaviour was attributed to the amide derivatives, although the bis (valine acid) derivative was also powerful. Contrary to mono-oxalamide derivatives, the derivatives with two amino acid–oxalamide units bridged with an aliphatic spacer showed potent gelation in oil only as ester derivatives. Due to the gelation results, the structural differences of the oxalamides as the main driving force for self-assembly and consequently gelation ability in oil will be published separately. The oxalamide supergelators with thixotropic properties in oil were subjected to further investigation and determination of possible applications. 

**Scheme 1:**
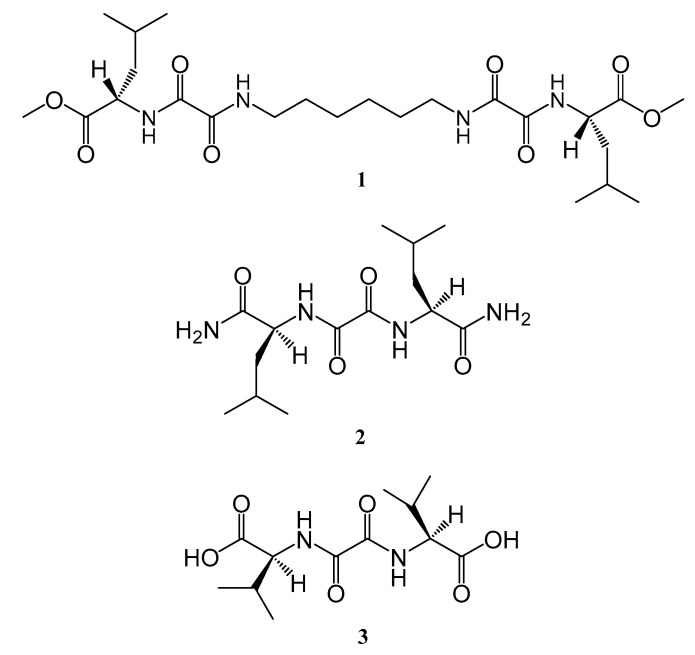
The structures of selected low molecular weight organogelators **1**, **2**, and **3**.

Three chiral oxalamide derivatives with terminal groups as ester (**1**), amide (**2**), and carboxylic acid (**3**) were selected for detailed examination (Scheme 1). The leucine and valine derivatives showed the most convenient gelation results with pronounced thixotropic properties. The other derivatives did not show or showed poor gelation capabilities or did not show thixotropic properties, which are mandatory for any of the possible applications. The applicability of supergelators in various oils for industrial applications without self-recoverable potency is not viable (Table 1 and Table S1). One of the key challenges in the field of organogelation is to find gelators that are cheap, easily available, thixotropic, and required in low concentrations to form a structured system.

We performed gelation experiments in sunflower oil and noticed unexpected behaviour of the compounds **1**, **2**, and **3** compared to the published gelation results of the selected mono and bis-oxalamide derivatives [12,43]. The gelators showed extraordinary gelation capability in oil and self-healing properties that were not related and published in the previous papers. 

The compounds **1**–**14** have been examined in a previous work as potential gelators of different organic solvents and water. The self-assembling principle of the oxalamide derivatives as a powerful hydrogen bonding driving unit was investigated in detail. The self-assembly of the oxalamide compounds and the influence of the different types of terminal groups were investigated using TEM, SEM microscopy, DSC calorimetry, FTIR, and NMR spectroscopy. Due to insufficient solubility of the compounds **2**–**11** in most of the common organic solvents except the most polar solvents, such as DMSO, DMF, MeOH, the gelation of solvent mixtures was investigated. The gelators showed poor gelation properties in the examined solvents. The compounds **1** and **12**–**14** showed versatile gelation abilities examined in different lipophilic solvents and solvent mixtures with very low gelation efficiency independent of the type of the terminal group (carboxylic acid, methyl ester) [43]. The thixotropic properties were not examined in the published papers. Contrary to the experiments performed in organic solvents, the bisoxalamide ester derivatives (**1** and **13**) showed outstanding gelation capacity in various vegetable oils. Despite that, the bisoxalamide acid derivatives (**12** and **14**) showed very poor or no gelation at all in vegetable oil. 

Gelator **1** showed very poor gelation capability in different organic solvents, solvent mixtures, water [43], and liquid crystals [45]. The lowest critical gelation concentration was 0.45 wt% in tetralin, and mostly the minimal gelation concentration was about 1 wt% in different solvents. Demonstrating the powerful gelation efficiency of gelator **1** in oils compared to the poor gelation properties in examined organic solvents and solvent mixtures, the minimal gelator concentration was 0.042 wt%. Regarding all the differences noticed in the gelation abilities of the compounds **1**–**14**, there is no decisive correlation of the powerful gelling ability in vegetable oil with the previous gelation results obtained in various organic solvents and solvent mixtures. 

**Figure 1:**
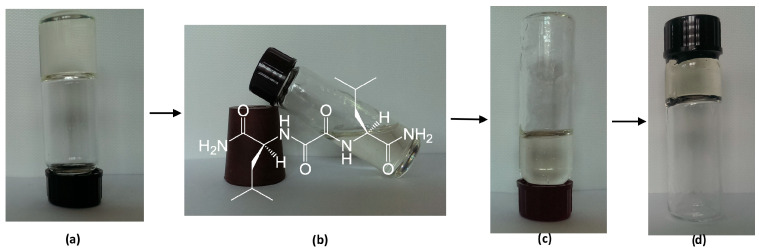
Visual observation of the thixotropic property of 0.1 wt% of gelator **2** in sunflower oil. (**a**) Gel formed from hot solution upon cooling; (**b**,**c**) low viscosity fluid formed by vigorous hand shaking; (**d**) gel reformed after standing for 5 min at room temperature.

It is also interesting to explore the influence of the oil type on the gelling properties of the compounds. The selected compounds which showed powerful gelation in sunflower oil continued to do so in the examination of gelation properties in soybean and olive oil. The maximum amount of a gelled volume per compound was determined (Table 1). The self-healing properties of the examined gelators were determined at a half of the maximum gelling volume. The formed gel was subjected to an external mechanical stress (shaking) till the gel was transformed into a sol state. The self-healing process (recovery to gel state) occurring after standing at room temperature was measured every 5 min by the tube inversion method and visual observation (Table 1). For example, 10 mg of the compound **2** (an amide derivative) was capable of causing solidification of 13.2 mL of sunflower oil, 27.2 mL of olive oil, and even 43.3 mL (0.026 wt%) of soybean oil. When treated with external mechanical stress (shaking), soybean oil gel **2** lost most of its viscosity and transformed into a sol; after resting for 5 min at room temperature, the gel completely regenerated. The self-healing process after the gel-to-sol transition, which was brought about by mechanical stress, can be repeated many times. Visual observation of the gel formation and self-healing of the oil gel are presented in Figure 1. 

The compounds **1** and **3** showed different gelation behaviours in various oils compared to the compound **2**, still with strong gelling potency (0.04 wt%–0.12 wt%). The consistency in the gelation capacity of the three structurally different chiral oxalamide gelators for a specific oil have not been noticed, indicating a strong influence of the gelator structure and solubility as the main prerequisite. The overall gelator efficacy in different oils was in the following order: acid < ester < amide derivative. In a previous examination, it was concluded that next to polarity, the viscosity of the oil also affected the self-assembly of the gelator molecules [46]. Additionally, the differences in the carbon chain length of the oil phase, the content of fatty acids, and the chemical composition have profound influence on the gelation properties [47]. 

The interplay between gelator–gelator and gelator–solvent interactions deeply depend on the chemical composition and the polarity of the oil. In a more polar environment, gelator–solvent interactions are more pronounced, mostly affecting the gel strength [48]. Contrary to the pronounced gelation efficacy noticed in olive oil, the self-healing properties were more pronounced in sunflower and soybean oil. This shows the impact of the oil composition, the ratio of monounsaturated fatty acids (MUFAs) and polyunsaturated fatty acids (PUFAs), and the presence of the small percentage of various polar components capable of interacting with the gelator molecules. Depending on the gelator structure, the synergistic effect of the oil composition clearly influenced the gelation capacity in certain cases. Nevertheless, the self-healing phenomena observed after the mechanical disruption of the gel network were also influenced by the oil polarity since gelator–solvent interactions play a key role in the re-establishment of broken fibre aggregates and 3D networks capable of entrapping oil and preventing it from flowing. Polar components in olive oil can cause the formation of a solvation layer or even interactions with gelator molecules and therefore inhibit gel reformation and gelator–gelator interactions, consequently causing slower gel recovery as is observed in olive oil gels.

The detailed examination of the influence of oil type, gelation capacity, and self-healing potential on the viscoelastic properties of the gels is explained in the next section. An excellent gelation property of the presented gelators in various oils is the apparent possession of an optimum balance between the affinity and insolubility in the oil phase. Therefore, a suitable ratio between gelator–gelator and oil–gelator interactions ensures the formation of a continuous 3D network of gelator molecules and subsequent solidification of the oil phase capable of reforming after mechanical disruption. After performing the detailed study presented in this paper, we can understand the powerful influence of the gelators’ structure, the self-assembly of the gelator molecules at a nano scale, the entanglement of the aggregates at a microscale, the 3D morphology capable of entrapping oil to a high extent, the influence of oil polarity and viscosity, the chemical composition of the oil phase, the oil–gelator interactions, and optionally the interactions with various bioactive molecules present in different compositions in various oils that altogether in a complex manner affect the gelation potency and the self-recovery of the investigated oil gels.

#### Stability of Oil Gels and Gelled w/o Emulsions

**Figure 2:**
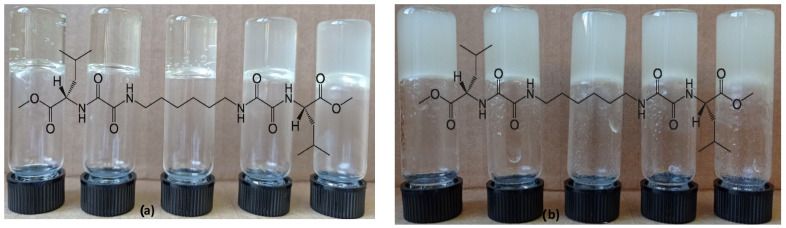
Stability of **1** (**a**) sunflower oil gels and (**b**) oil gelled w/o emulsion (10% H_2_O, *v*/*v*) in concentrations of 1.44 mmol/L; 2.0 mmol/L; 4.3 mmol/L; 7.6 mmol/L; and 12.0 mmol/L in ascending order.

The bis (leucine) bisoxalamide ester derivative with a hexamethylene carbon chain between two oxalamide units (**1**) showed the profile of potentially the most applicable gelator. Gelator **1** is a supergelator in different vegetable oils with exceptional self-healing properties and desirable solubility properties. The compound **1** is not soluble in water. Additionally, gelator **1** is capable of being a gel oil in the presence of small amounts of water molecules in the form of oil gelled w/o emulsion (Figure 2b). Different concentrations of **1** sunflower oil gels that are stable for years are presented in Figure 2a. It could be seen that **1** gels are mainly transparent gels, and turbidity was observed only at very high concentrations (12 mmol/L). An addition of 10% *v*/*v* of water in **1** sunflower oil gels caused the formation of opaque gels (Figure 2b). The gelled emulsions were stable for months (Figure S8, Supplementary Material).

The **2** sunflower oil gel withstood a very small amount of water molecules and showed unstable properties with time. The oil gel of the compound **3** immediately precipitated after the addition of 10% water and the subsequent heating–cooling procedure. The compounds **2** and **3** are acid and amide derivatives, respectively. As reported previously in the paper of Makarević et al., besides the main oxalamide hydrogen bonding units, the terminal amide or acid groups provide a certain contribution in the formation of additional hydrogen bonds in the formation of gel fibres [12]. An addition of water molecules in stable and transparent oil gels causes competition for hydrogen bondings and consequently prevents the formation of gel fibres and gel network. Contrary to the observations with acid and amide gelators, gelator **1** as an ester derivative showed high tolerance for even 30% water content in oil gels. Due to the aforementioned facts, the gelation properties of gelator **1** were explored in detail.

### 2.2. TEM Investigation

**Figure 3:**
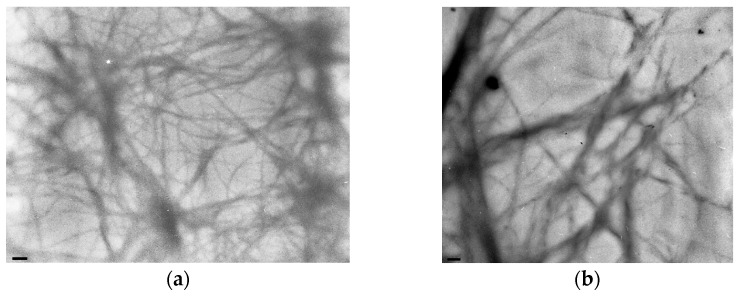
The TEM image of (**a**) **1** sunflower oil gel showed the existence of fibres and ribbons with diameters of 60–200 nm, (**b**) **1** oil gelled w/o emulsion (10% H_2_O, *v*/*v*) showed the existence of some fibres and mostly twisted ribbons with diameters from 100 nm to 1 μm (bar = 1 μm).

TEM micrographs of investigated gelators showed distinct morphologies depending on the solvent used. The morphologies of **1**, **2**, and **3** gelators in sunflower oil were monitored using TEM microscopy Figure 3, Figures S1 and S2). The TEM image of **1** sunflower oil gel showed the existence of fibres and ribbons with diameters (d) of 60–200 nm (Figure 3). TEM micrographs of **2** sunflower oil gel showed the existence of fibres and ribbons with d values of 100–400 nm and of **3** sunflower oil with 40–400 nm (Figure S1). The structurally different chiral oxalamide gelators showed the existence of self-assembled aggregates in a similar range of widths in sunflower oil. The addition of water in **1** oil gel causes the formation of broader twisted ribbons with widths of 100 nm–1 μm, with a higher extent of ribbons in **1** gelled w/o emulsion compared with that in **1** oil gel (Figure 3).

The TEM image of the **1** tetralin gel showed the formation of fibres with d values 8–20 nm (Figure S2b). The tiniest aggregates of gelator **1** were observed in tetralin, as it was the most lipophilic of the used solvents; whereas with an increase in the solvent polarity (tetralin < sunflower oil < DMSO/water), the quantity of ribbons increased along their widths. This observation could be explained by the unfavourable aggregate–solvent interactions (aggregate solvation), which in the case of polar solvents, induces the formation of wide ribbons to decrease the lipophilic surface of the aggregates exposed to polar solvents. Gelator **1** in DMSO/water gel self-assembles into wide ribbons with an apparent twist (80–200 nm and even to 600 nm widths; Figure S2a)). The solvation effect has been also noticed in **1** oil gelled w/o emulsion, as the formation of wide twisted ribbons till 1 μm is observed. The gels in the DMSO/water mixture and gelled w/o emulsions are opaque gels. The chiral oxalamide gelators showed ambidextrous gelation ability in various solvent mixtures, such as the gelation of water/DMSO mixtures, oils, and much less polar organic solvents. The proper balance of hydrogen bonding sites and the spatial arrangement of lipophilic groups in the oxalamide derivatives enables different organisations in water, mostly governed by lipophilic interactions, and in organic solvents by intermolecular hydrogen bonding [43].

### 2.3. Thermal Stability of Gels: DSC Study and Gel–Sol Phase Transitions

**Figure 4:**
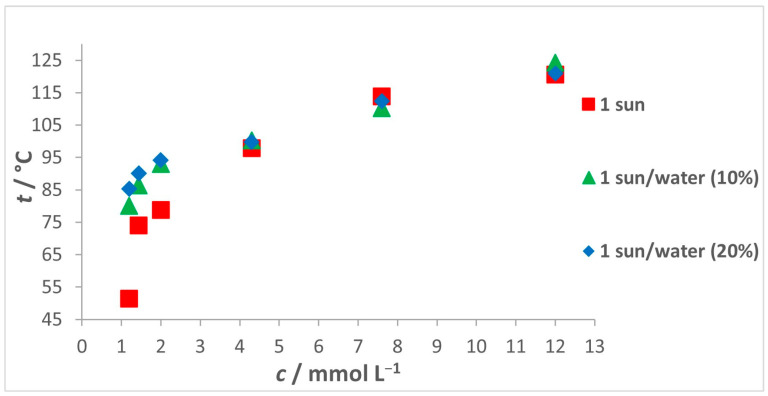
Gel–sol phase transition diagrams of **1** sunflower oil gels (red), **1** oil gelled w/o emulsions (10% H_2_O; *v*/*v*; green), c) **1** oil gelled w/o emulsions (20% H_2_O; *v*/*v*; blue) obtained by the dropping ball method.

To study the thermostability of the gels, gel-to-sol phase transition temperatures (T_g_) of the **1** oil gels and **1** oil gelled w/o emulsions (10–20% H_2_O, *v*/*v*) at different gelator concentrations were determined by the dropping ball method (Figure 4 and Table S2). The gel–sol phase transition curves showed a rise in the gel melting temperatures with an increase in the concentration of the gelator. 

For the **1** oil gels, the gel–sol phase transition curve shows an increase in the melting temperatures up to 0.012 mol/L that does not still level off. The gel–sol phase transition curves of **1** oil gelled w/o emulsions (10% and 20% H_2_O) follow the gel–sol phase transition curve of **1** oil gel at concentrations higher than 0.0043 mol/L. The gel–sol phase transition curves of gelled emulsions at gelator concentrations lower than 0.0043 mol/L showed much higher T_g_ temperatures compared to that in **1** oil gel. The gel–sol phase transition temperatures of gelled emulsions with a higher extent of water (20%) showed even higher T_g_ temperatures at lower examined gelator concentrations. The influence of the water content on the self-assembling properties of gelator **1** in sunflower oil at lower gelator concentrations can be clearly seen (Figure 4). It can be assumed that gelator molecules present at lower concentrations are completely involved in the self-assembling process, producing a defined gel network of certain thermostability. Addition of water molecules induces a different type of organisation of gelator **1** due to hydrophobic interactions, causing formation of a gel network with higher thermostability. It is interesting to observe that much denser networks formed at much higher gelator concentrations showed small differences in the thermostability of oil gels and emulsion gels. The results of DSC studies obtained at 0.045 mol/L can be extrapolated with the phase diagrams, resulting in good corelation with the results obtained by the dropping ball method.

The gel–sol phase transition temperature of 51 °C determined for the gelator **1** concentration of only 0.06 wt% showed favourable results of T_g_ temperatures, offering the possibility to use a very low concentration of organogelator as a potential thermostable heat-resistant system. The melting temperatures (T_m_) and melting enthalpies (ΔH_m_) determined for **1** oil gel and **1** gelled w/o emulsions at 2.5 wt% obtained by the DSC study are shown in Table 2. The gel melting enthalpy change (ΔH_m_) measured for **1** oil gel at the transition temperature T_m_ = 141.6 °C is 17.86 kJmol^−1^. The measured gel melting enthalpy changes in the **1** oil gelled emulsions with 10% water and 20% water content are 36.85 kJmol^−1^ and 46.03 kJmol^−1^, respectively. 

The measured gel melting enthalpy change in the **1** gelled emulsion with 10% water exhibited a twofold higher ΔH_m_ value compared to that in the gel in sunflower oil. With an additional increase of water content, a further increase in the gel melting enthalpy change was observed. 

In agreement with this observation, the TEM images in Figure 3 show the presence of different polymorphic aggregates in oil and w/o emulsion gels due to solvation and the hydrophobic effect. In **1** gelled w/o emulsion, formation of twisted ribbons till 1 μm in comparison with **1** oil gel fibres and ribbons with widths till 200 nm have been observed. Consequently, formation of wider ribbons resulted in higher gel melting enthalpy changes. 

As shown by the results discussed in the paper of Šijaković et al. [43], the intermolecular hydrogen bonding between oxalamide units of **1** represents the major contribution to the overall stabilization of gel aggregates in lipophilic solvents, which may also exist in the DMSO/water gels. However, in the latter solvent system, the stabilizing contribution of the intermolecular lipophilic interactions between *i*Bu groups of Leu may become more important than hydrogen bonding as demonstrated by van Esch et al. for another type of gelator [49]. The results of DSC studies clearly showed that ΔH_m_ values for gels with solvents of different polarities cannot be explained by the lipophilicity relationship due to their bolaamphiphilic structure and the formation of gels in highly polar and highly lipophilic solvents [43]. The gel melting enthalpy changes depend on different factors including solubility and strengths of hydrogen bonding and lipophilic interactions involved in the stabilization of the gel aggregates in oil and gelled w/o emulsions [50]. Depending on the gelator concentration, the temperatures of gel–sol transitions had a wide range of T_g_ (51–142 °C), which could be favourable for possible applications, especially in preventing oil mobility, since the low molecular weight organic gelators possess thermoreversible properties. 

### 2.4. Oscillatory Rheology of Oxalamide Oil Gels

The mechanical properties of the gels result from the properties of the self-assembled fibres, their thickness, length, the number and type of no-covalent interactions, and their spatial distribution. All investigated oil gels exhibited a non-Newtonian shear-thinning behaviour characterized by a decrease in viscosity with increasing shear rate.

#### 2.4.1. Amplitude Sweep

**Figure 5:**
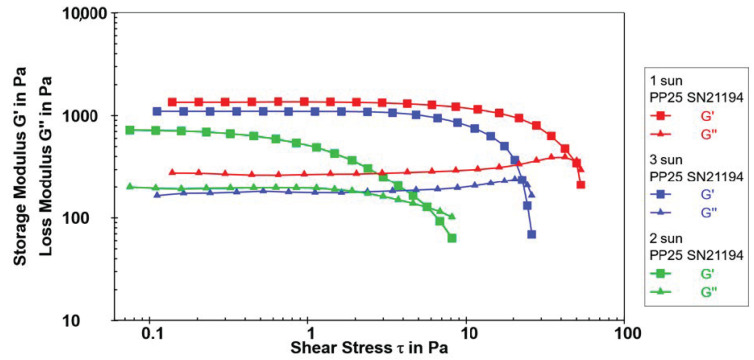
Amplitude sweep tests (G′ and G″ values) of 0.2 wt% of **1**, **2,** and **3** sunflower oil gels examined at 15 °C.

**Figure 6:**
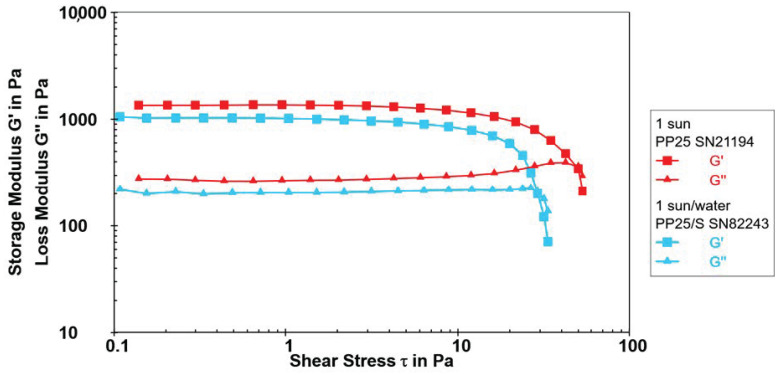
Amplitude sweep tests (G′ and G″ values) of 0.2 wt% of **1** oil gel and **1** gelled w/o emulsion (10% H_2_O; *v*/*v*) at 15 °C.

The storage modulus G′ (Pa) represents the elastic portion of the viscoelastic behaviour, which describes the solid state behaviour of the sample. Typically, for gels, the elastic component dominates (G′) over the loss modulus or viscous component (G″) at small applied shear and attains a plateau in the linear response region (LVR or linear viscoelastic region). The linear viscoelastic region covers the range wherein the applied stress does not considerably affect the 3D structure of the gels. The point wherein the linear relationship between stress and strain is disturbed is referred to as the yield point. From this point on, the gel structure is being changed. Strain (γ) sweep tests were performed for gelators of 0.2 wt% in the strain range from γ = 0.01 to 100% at an angular frequency ω = 10 rad s^−1^ at 15 °C. The G′ and G″ values remained approximately independent of the applied strain up to 0.1%. Amplitude sweep results are presented in Figure 5. The storage modulus (G′) values (determined at LVR) of sunflower oil gels containing 0.2 wt% of the gelator **1, 3**, and **2** are 1371 Pa, 1102 Pa, and 709 Pa, respectively (Figure 5 and Table 3). The storage modulus (G′) value of 0.2 wt% of **1** gelled w/o emulsion (10% H_2_O) is 1031 Pa (Figure 6).

The yield stress values observed for the sunflower oil gels containing 0.2 wt% of the compounds **1**, **3**, and **2** are 22.2 Pa, 12.4 Pa, and 1.2 Pa, respectively. The flow point values for the sunflower oil gels containing 0.2 wt% of the compound **1**, **3**, and **2** are 48.4 Pa, 22.7 Pa, and 5.7 Pa, respectively. The loss factor, tan δ, measured at a constant temperature and at a specified frequency, is introduced as a parameter characterizing the damping properties of the materials. The loss factor is defined as the ratio of energy dissipated in the material during vibrations to the maximum potential energy stored in the material, presented as the ratio of shear moduli.

The rheologically determined quantity loss factor (tan(δ) = G″/G′) determines the relative elasticity of the viscoelastic materials. The gels formed in the investigated oils showed relatively low tan(δ) values in a range from 0.16 to 0.27, which is indicative of a relatively high elastic modulus for the gels in almost all examined vegetable oils. The gels with a value of tan(δ) = 0.1 belong to stiff gels. The stiffest oil gel is the **3**-oil gel with the loss factor 0.16. Gelator **1** showed the most common relative elasticity value for low molecular weight gelators (tan(δ) 0.19). A somewhat weaker organisation was obtained for oil gelator **2**. For the **2** oil gels, the yield point and flow point values have been obtained at considerably lower stress and strain values compared to **1** and **3** gels. The existence of broad ribbons (100–400 nm) in **2** oil gel could be the reason for the lower mechanical strength of the gel network, consequently forming a tenuous gel network at the examined concentration. Considering the gel efficiencies presented in the gelation experiments, gelator **3** showed the lowest gelation efficiency in sunflower oil compared to **1** and **2**. Contrary to that, gelator **3** at an examined concentration of 0.2 wt% showed the highest relative elasticity and structural organisation, devoted to a powerful self-assembly of acid oxalamide derivatives. Nevertheless, the ester derivative **1** showed almost a twofold higher yield and flow point values and higher G′ values compared to the **3** oil gel. However, **1** oil gels have a lower relative elasticity compared with **3** oil gels and consequently could have different possible applications.

The formation of **1** oil gels in the presence of water have been examined by TEM and DSC studies, revealing a deep influence of the added water on the gel structure. The rheological investigation additionally supported the beforementioned observations. The **1** gelled w/o emulsion showed somewhat lower elastic moduli and similar relative elasticity but a twofold lower yield point and flow point values compared to pure **1** oil gel. The yielding zones of **1** oil gel and **1** gelled w/o emulsion were 26 Pa and 16 Pa, respectively. The significant difference in flow point values of **1** oil gel and **1** gelled w/o emulsion were 48.4 Pa and 28.7 Pa, respectively. The oscillatory sweep results showed the powerful influence of a small percentage of water on the formation of different aggregates and consequently weaker viscoelastic parameters of the formed gel network. A lower yielding point value, shorter yielding zone, and a plateau of G″ in the area before transition of a gel to sol state (G′ = G″) provided insights into the morphological changes of the gel network in the emulsion compared to the pure oil gel, as evidenced with microscopic and DSC techniques. The **1** oil gelled w/o emulsions could have a potential application as a substitute in low fat products overall.

#### 2.4.2. Frequency Sweep

**Figure 7:**
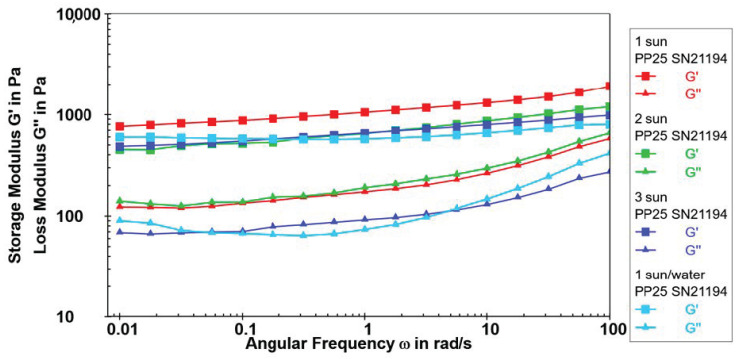
Frequency sweep tests (*G*′ and *G*″ values) of 0.2 wt% **1**, **2,** and **3** sunflower oil gels and **1** gelled w/o emulsion (10% H_2_O, *v*/*v*) performed at 0.1% strain at 15 °C.

Frequency sweep studies (ω = 0.01–100 rad/s at 0.1% strain) of the gels at 15 °C indicate that the storage modulus (G′) and loss modulus (G″) values are mostly independent of the applied frequency within the linear viscoelastic regions (LVRs) (Figure 7). All investigated compounds in the form of gelled oils (**1**, **2,** and **3**) and **1** gelled w/o emulsion according to the rheological measurements are true gels stable for a prolonged period of time. Frequency sweep for gels containing 0.2 wt% of the compound **1** showed a lower relative elasticity in gelled w/o emulsion at higher frequencies (100 rad/s, loss factor 0.5) compared with the loss factor determined for pure **1** oil gel (100 rad/s, loss factor 0.3). The loss factors at a frequency of 0.01 rad/s for the **1** oil gel and the **1** gelled w/o emulsion were about 0.1. The low molecular weight organic gelators showed a sensitivity noticed at higher frequencies due to interactions between smaller gelator aggregates and dissolved gelator molecules contributing to higher G″ values. This behaviour observed in the frequency sweep was characteristic for the most of investigated gelators. Frequency sweeps at very low frequency values (0.01–0.1 rad/s) were performed to evaluate a gel material at rest, during which different oil gels and gelled w/o emulsion showed exceptional stability.

#### 2.4.3. Thixotropic Properties

**Figure 8:**
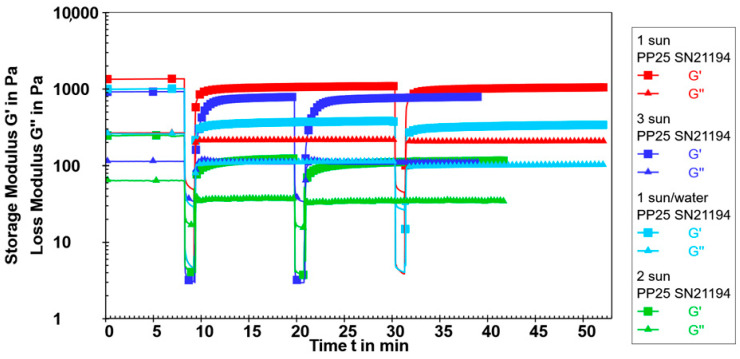
3-interval thixotropy test (3ITT) (G′ and G″ values) of 0.2 wt% of **1**, **2,** and **3** sunflower oil gels and **1** gelled w/o emulsion at 15 °C. Linear viscoelastic region (LVR): strain = 0.1%, frequency = 10 rad/s; destructive region (DR): strain = 100%, frequency = 10 rad/s; recovery region: linear viscoelastic region (LVR): strain = 0.1%, frequency = 10 rad/s.

The possibility that the gelators of different vegetable oils and gelled w/o emulsion mentioned in Table 1 are thixotropic was explored by the 3ITT test. In the thixotropic experiments, rheological measurements were conducted on the fresh gels at 15 °C under initial conditions at which they were in their linear viscoelastic regimes (a strain of 0.1% and angular frequency of 10 rad/s) for 500 s to establish baseline values for G′ and G″. In the performed studies with oil gels, we have observed viscoelastic recovery after the cessation of destructive strain (Figure 8). 

Applied conditions in the 3-ITT test for the first 500 s were strain = 0.1%, angular frequency = 10 rad/s; for the next 60 s, strain = 100%, angular frequency = 10 rad/s; thereafter, strain 0.1% and angular frequency = 10 rad/s. Figure 8 shows the evolution of G′ and G″ for the 0.2 wt% **1**, **2,** and **3** sunflower oil gels and **1** gelled w/o emulsion (10% H_2_O; *v*/*v*) after applying 100% strain, a condition that leads to the loss of its viscoelasticity. After that, the original conditions were reapplied in order to monitor the recovery of the viscoelastic properties of the gels. 

All investigated gelators showed self-healing properties in different quantities. The fastest recovery was observed for **1** sunflower oil gel (67%), then **3** oil gel (52.7%) in the first 60 s (Table 4). After 10 min, the recovery of **1** oil gel viscoelasticity was 80.1% and of **3** oil gel 84.8%. The **2** oil gel showed a recovery of 50% and **1** gelled emulsion only 37%. Addition of water in **1** oil gel caused the formation of different aggregates and consequently a morphology of the gel network in the gelled w/o emulsion incapable of total self-recovery after mechanical agitation (Figure S3). 

The recovery of G′ and G″ values in two consecutive thixotropic recovery cycles was investigated (Figure 8 and Table S3). After re-application of the 100% destructive strain in the fourth cycle, a recovery higher than 90% of the previous G′ values of the investigated gels were observed. The self-assembled network of the gels is stabilized by intermolecular H-bonding interactions. The H-bonding interactions have been reported as the main driving force responsible for the reassembly of fibres in the 3D gel network after cessation of a destructive shear [17]. The oxalamide bridge as a hetero-functional unit is responsible for the unidirectional self-assembly of molecules in different solvents. In addition, the terminal amide and acid groups could have a favourable contribution on viscoelasticity and viscoelastic recovery in oil gels after the cessation of mechanically applied destructive force. Poor viscoelastic recovery (37%) of **1** gelled oil emulsion (10% water) supports the theory. Water molecules present in the system after disruption of the gel network were in competition for H-bonding units, consequently preventing the complete reassembly of the fibres into the 3D gel network. The self-healing properties of the oil gels determined in the 3ITT consecutive investigation are proof of the self-recoverable properties of the distinguished LMWOG as potential soft materials for diverse applications in food, cosmetics, and pharma industry. The examples of controlled delivery of active substances and use of oil gels as fat substitutes in food products will be explained in the next chapters. 

### 2.5. Influence of the Oil Type on Rheological Properties of the Gels

The nature of gelator molecules predominantly determines the self-assembly motifs, the microstructural and viscoelastic properties of organogels. Nevertheless, it has been determined to be a strong influence along with the oil type on the rheological properties of oleogels. One of the interesting oleogel mixtures formed by the self-assembly of γ-oryzanol and β-sitosterol into tubules has been affected by the polarity of the oil [25]. In further investigation, researchers concluded that, next to polarity, the viscosity of the oil also affected the self-assembly of gelator molecules [41]. Increasing oil viscosity or decreasing oil dielectric constant, firmness, and rheological parameters linearly increased as published in the paper regarding monoglyceride oleogels [51]. Furthermore, the researchers mentioned the ability of some oils to make dipole–dipole rotation and that the high solubility of monoglycerides in castor oil has further influence on structuration. Regarding the difference in carbon chain of the oil phase, long chain triglyceride-based organogels were stronger than medium chain triglyceride-based organogels during network formation [52]. Overall, gel strength decreased when the interactions between the gelator molecules and the oil increased. The interactions deeply depended on the chemical composition and the polarity of the oil. One of the examples was observed in ricinelaidic acid-based oleogels, where the gelator molecules were able to form hydrogen bonds with moieties on oil and subsequently caused a decrease in the gelation efficiency [53]. The results presented in the paper by E. Scholten et al. [48] showed that the gel strength of the network formed by protein aggregates is affected by the polarity of the oil, resulting in weaker gels in more polar oils because of larger particle–solvent interactions.

The linear viscoelastic material functions of montmorillonite-based oleogels were found to depend on the content of fatty acids in the vegetable oils. Oleogels formulated with vegetable oils containing high SFAs/MUFAs and low UFAs/PUFAs contents achieved higher plateau moduli as a consequence of stronger microstructural networks [42]. Further, they reported that oleogels formulated with linseed and olive oil showed poor structure recovery properties.

Three different oils were used in the current study. Sunflower oil and extra virgin olive oil were purchased at a local supermarket and used without purification. Soybean oil was purchased from Alfa Aesar. The declared composition of the sunflower oil was 12% saturated fatty acids, 27% monounsaturated fatty acids, and 61% polyunsaturated fatty acids. The soybean oil consisted of 15% saturated fatty acids, 22% monounsaturated fatty acids, and 57% polyunsaturated fatty acids. The extra virgin olive oil consisted of 12% saturated fatty acids, 68% monounsaturated fatty acids, and 9% polyunsaturated fatty acids. Sunflower oil is a refined oil with minor polar components, with the exception of tocopherols. Extra virgin olive oil can contain a substantial amount of 1–2% of polar molecules such as polyphenols, phosphatides, pigments, and sterols [54]. Due to the difference in the oil composition and the oil polarity, we expected different influences on the gelation properties of the oxalamide compounds. 

**Figure 9:**
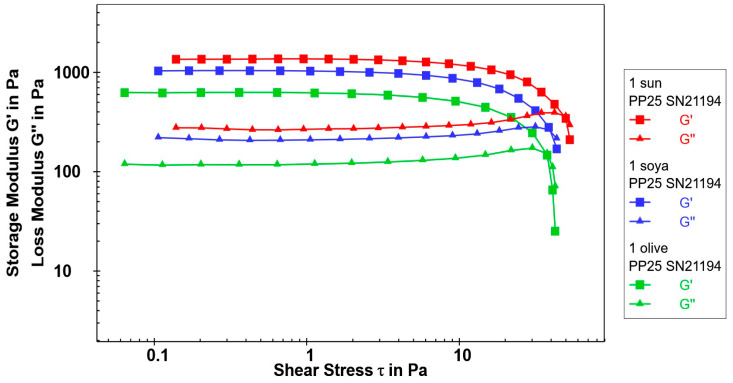
Amplitude sweep tests (*G′* and *G″* values) of 0.2 wt% of **1** sunflower oil gel (1 sun), **1** soybean oil gel (1 soya), and **1** olive oil gel (1 olive) performed at 10 rad s^−1^ and 15 °C.

Using different oil types for structure formation by oxalamide **1** gelator, G′ increased as olive oil > soybean oil > sunflower oil, which indicated a higher amount of gelator–solvent interactions in a more polar environment, causing weaker gel strength (Figure 9 and Table 5). The higher content of polar molecules in olive oil favoured gelator–solvent interactions, forming weaker gels.

Regarding the gelation properties of gelator **1** (Table 1), it could be concluded that the gelation efficacy (maximum gelation capacity) is similar in sunflower oil and soybean oil, but more pronounced in olive oil (more than 30%). Contrary to the maximal gelation efficacy noticed for olive oil, the self-healing properties were more pronounced in sunflower and soybean oil. Polar components in olive oil can cause formation of a solvation layer or even a co-assembly or interactions with gelator molecules and therefore inhibit gel formation and gelator–gelator interactions, influencing also the self-healing properties of gels, as evidenced in the inverted test tube experiments and 3ITT rheological tests. 

Most of the investigated gels in sunflower oil, soybean oil, and olive oil were moderately stiff gels even at a low concentration of 0.2 wt%. The highest G′ value (1372 Pa), yield point (22.2 Pa), and flow point (48.4 Pa) values were noticed for **1** gel in sunflower oil. The lowest values were determined for **1** olive gel with G′ value (628 Pa), yield point (14.1 Pa), and flow point (37 Pa) values. The **1** olive oil gel has the lowest G′ modulus, but still has comparable shear stress values with rheological data determined in different oils with much higher deformation variables (shear strain at flow point 16.8%). The relative elasticity (0.19–0.20) is very similar for all examined oil gels. The yielding zone had the longest interval for **1** sunflower oil gel and very similar values for soybean and olive oil. 

**Figure 10:**
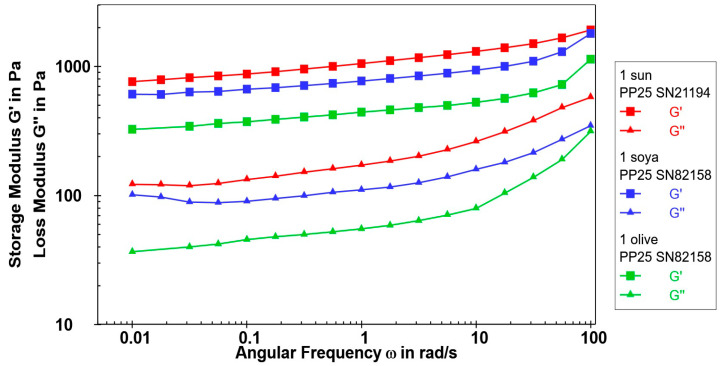
Frequency sweep test (G′ and G″ values) of 0.2 wt% of **1** sunflower oil gel (1 sun), **1** soybean oil gel (1 soy), and **1** olive oil gel (1 olive) at a strain 0.1% and 15 °C.

Frequency sweep studies for different oil gels containing 0.2 wt% of the compound **1** showed consistent curves with G′ > G″ values in the examined area (Figure 10). All examined gels showed somewhat higher values of G′ and G″ at higher frequencies, which have been characteristic of most of the investigated low molecular weight organic gelators, especially at lower concentrations. Frequency sweeps at very low frequencies (0.1–0.01 rad/s) were examined to evaluate oil gels at rest, during which the oil gels showed exceptional stability independent of the oil type used. The prolonged stability of oil gels is a very important property due to the potential product shelf-life. 

**Figure 11:**
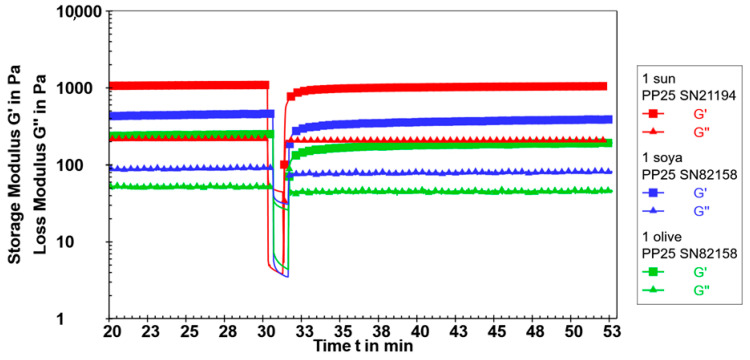
3-interval thixotropy test (3ITT) (G′ and G″ values) of 0.2 wt% of **1** sunflower gel (1 sun), **1** soybean oil gel (1 soy), and **1** olive oil gel (1 olive). Linear viscoelastic region (LVR): strain = 0.1%, frequency = 10 rad/s; destructive region (DR): strain = 100%, frequency = 10 rad/s; recovery region: linear viscoelastic region (LVR): strain = 0.1%, frequency = 10 rad/s.

Besides the formation of elastic networks, properties such as resistance against structure breakdown and structure recovery are important aspects for different applications. Figure 11 shows the rheological behaviour of the oleogels as a function of the applied strain. Firstly, strain value determined in the linear viscoelastic region (where G′ is independent on the applied strain) was applied and after that increased strain amplitudes (large strains) caused deformation of a network. To further assess the ability of the network to restore after deformation is applied, we examined the structure recovery over time at low deformation. After subjecting the different gels to large deformations (100%) to induce structure breakdown, the strain was reduced to 0.1% (LVR range) to examine the reformation of network junctions or thixotropic behaviour over time. The recovery of G′ was followed for 30 min and the results are presented in Table 6. During recovery, the examined gels reformed for which G′ > G″. 

All investigated gelators in different oils during the 3ITT tests showed self-healing properties corresponding to the results performed by visual observation in test tubes after mechanical agitation (Table 1). The fastest recovery was observed for **1** sunflower oil gel (95%), then **1** soybean oil gel (81.5%). The slowest recovery in the first 60 s and recovery after 15 min is 75% for **1** olive oil gel (Table 6). The presented results are in good accordance with the results observed by the test tube inversion method. The results indicate that **1** sunflower oil gel had the most prominent rheological parameters regarding the amplitude sweep, frequency sweep, and thixotropy test. In comparison to the determined rheological measurements, it is interesting to notice that gelator **1** showed lower gelation efficiency in sunflower oil compared to a much higher efficiency determined in olive oil. 

In accordance with the gelation capacity and self-recovery of the examined gels, it is convenient to conclude that the physical properties observed in sunflower and soybean oil correspond to oil composition similarities, contrary to a somewhat weaker recovery observed in olive oil due to different chemical composition (mostly monounsaturated fatty acids and 1–2% polar components). The behaviour of gelator **1** in olive oil is in accordance with previously published results as explained at the beginning of this section. This result can be related to the affinity of gelator molecules for the solvent. The change in oil type resulted in a difference in the gelator–gelator and gelator–solvent interactions, which affected the rheological properties. For gelator **1**, an increase in oil polarity resulted in a decrease in G′ due to more favourable gelator–solvent interactions. We assume the polar components in olive oil, amongst other factors, influence the higher gelator–solvent interactions and consequently result in weaker gel strength and slower recovery. Although, attention should be paid to the higher gelation efficiency observed in olive oil for diverse oxalamide derivatives. 

If there was low affinity for the solvent, gelator–gelator interactions will dominate over gelator–solvent interactions in oil. Strong gelator–gelator interactions give rise to faster recovery of G′ upon lowering the strain. Figure 11 shows the amount of G′ recovery for different oil types after 30 min, defined as the G′ after specified recovery time divided over the initial gel strength. The ability of oil gels to flow under large deformations and regenerate an elastic network after the deformation is reduced offers wide benefits to a variety of food and medicine applications. 

The physical properties of oleogels amongst others depend on the properties and spatial arrangement of hydrocarbon chains in the fatty acid composition. Olive oil, contrary to sunflower and soybean oil, contains more than 68% monounsaturated fatty acids, mainly oleic acid. Saturated fatty acids, for example, stearic acid molecules have straight chain structure, while oleic acid chain structure is bent. Spatial arrangement factors may be one of the reasons for the differences in gelation efficiencies and self-healing properties of structurally different gelator molecules. The composition of olive oil is primarily triacylglycerols (~99%) and free fatty acids, mono- and diacylglycerols, phenols, and an array of lipids such as hydrocarbons, sterols, aliphatic alcohols, tocopherols, and pigments. Fatty acids present in olive oil are palmitic (C16:0), palmitoleic (C16:1), stearic (C18:0), oleic (C18:1), linoleic (C18:2), linolenic (C18:3), myristic (C14:0), heptadecanoic, and eicosanoic acids are found in trace amounts.

Stronger gels were produced when oils rich in more highly unsaturated fatty acids were used. It was suggested that the greater degree of conformational freedom associated with higher unsaturation leads to a higher molar volume of the solvent, which potentially facilitates the formation of a greater number of interpolymer junction zones, thus producing a stronger gel [55].

Due to the presented results, a distinct influence of the gelator structure, capable of forming unique aggregates and potentially additional no-covalent interactions with a small percentage of different bioactive molecules present in various oils, could be assumed. It is important to realize that the gelator molecules in the conducted experiments have been present in concentrations lower than 0.2 wt% and various bioactive compounds represent even 1–2% of oil composition depending on the oil type [56]. The potential interactions between specified gelator molecules and different bioactives in oil should be examined to determine a possibility of co-assembly with gelator molecules, self-sorting mechanisms, or interplay as additives contributing to higher/lower gelation efficiencies and self-recovery of a gel network [57]. As we have mentioned in the previous section, there are so many different factors such as fatty acid content, polarity, and the viscosity of the vegetable oils used that could influence the gelation and physical properties of various oil gels. These factors influence the gelator–oil interactions and as a consequence dictate the formation of a weaker or stronger gel network. Certainly, the influence of gelator structure in a wide range of oils should be investigated in detail. 

### 2.6. Controlled Release Properties of Oil Gels

Pharmaceutical drugs with low water solubility have received great attention within the scientific community. Physical (supramolecular gels) showed important properties such as injectability, stimuli responsiveness, and ease of synthesis. Most of the investigated systems were hydrogels [58]. They are widely used in biomedicine, especially for tissue engineering and drug delivery [59]. The low molecular weight organic gelators represent a class of compounds capable of encapsulating different amounts of active substances. Self-assembly of organic molecules enables the formation of nanostructured aggregates only through no-covalent bonds and formation of gel network of different morphologies. Factors that determine controlled release properties of gel media are gelator structure, concentration, morphology, solvent choice, solubility of bioactive substances, diffusion rate, and temperature. In the last 20 years, a lot of papers about controlled release properties of physical gels have been published. The various structures of LMWOGs have been used for drug delivery applications. The oil gels represent an interesting delivery system since the lipophilic drugs and bioactives are soluble in different oils [60]. Especially interesting would be the gelled w/o emulsion capable of entrapping hydrophilic and lipophilic drugs at the same time, controlling a release profile of both drugs.

**Figure 12:**
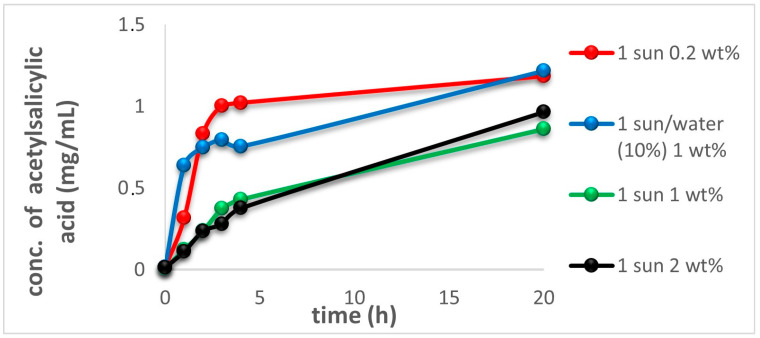
Controlled release profile of acetylsalicylic acid from **1** sunflower oil gels at different gelator concentrations (0.2 wt% red; 1 wt% green; 2 wt% black) and **1** gelled w/o emulsion (10% H_2_O; 1 wt% blue) at 25 °C.

To investigate a release profile of the selected analytes from prepared gels, a simple diffusion test using a reverse phase LC–MS method for determination of *acetylsalicylic acid* (AS), *ibuprofen* (IB)*,* and *hydrocortisone* (HC) was developed (Supplementary Material). Acetylsalicylic acid and ibuprofen are used as nonsteroidal anti-inflammatory drugs (NSAIDs) and hydrocortisone as a corticosteroid, widely used in the treatment of different diseases. The selected oil gels with the addition of the drugs were prepared in a well-closed glass vial and stored at room temperature. The gel surface was overlaid with 2 mL of MiliQ water (or ethanol in the case of ibuprofen and hydrocortisone monitoring) and a released profile of the investigated drugs was monitored regarding a protocol described in the section Materials and Methods. Considering all the results obtained through the gelation experiments, calorimetry, microscopy, and rheology, we decided to investigate in detail the drug delivery of gelator **1** in sunflower oil. Figure 12 presents the concentration driven controlled-release curves of acetylsalicylic acid depending on the concentration of gelator **1** in sunflower oil. 

The **1** sunflower oil gels as drug carriers were stable in contact with the water layer and stayed unchanged during the 24 h of monitoring. It is evident that the concentration of gelator **1** had a tremendous influence on the controlled release profile of acetylsalicylic acid. All examined drug delivery systems showed the existence of a logarithmic curve with a relative plateau obtained after 4 h. The lowest examined **1** gelator concentration (0.2 wt%) showed a steep slope of the curve and the fastest release of AS during the first 4 h, contrary to the results obtained for higher gelator concentrations (Figure S4). The 0.2 wt% of **1** oil gel released 93% of AS in the first 4 h (Figure 12). The **1** wt% of **1** oil gel released only 40% of AS in the same period of time. The maximal capacity of gelator **1** (plateau) was reached at ~1 wt%, since the 2 wt% of **1** oil gel released 37% of the examined drug in 4 h. Addition of water in oil gel causes the formation of a different gel network morphology, and water droplets in gelled w/o emulsion support a faster diffusion rate of the entrapped drug (AS) to the surface water layer. That is evident from controlled release curves; after 4 h, the **1** oleogelled emulsion (10% H_2_O, 1 wt%) compared to **1** oil gel (1 wt%) released 65% and 40% of AS, respectively (Figure 12 and Figure S4). 

Nevertheless, the gelator structure has a profound influence on the controlled release drug profile. Gelator **2** (amide derivative) was examined at 0.2 wt%. Contrary to **1** oil gel of the same concentration (release of 93% of AS), the **2** oil gel (0.2 wt%) showed slower release properties and in the first 4 h released 64% of AS. Gelator **3** is a diacid gelator whose oleogel is not completely stable (water triggers a dissolution of the gel and precipitation of the compound **3**) in contact with water during 24 h monitoring. However, after 3 h, gelator **3** at 0.2 wt% performed 100% release of AS and **1** wt% of **3** oil gel 84%. If we compare sustained release properties (64–100%) of the three structurally different oxalamide gelators (**1**, **2,** and **3**) examined at the same concentration (0.2 wt%) in sunflower oil, one can observe a deep influence of the gelator structure, consequently a self-assembly at nanoscale, gel morphology, a maximum gelling capacity, and an influence of viscoelasticity of 3D gel network at macroscale. Comparing gelation capacity, gel network morphologies, and viscoelasticity of **1**, **2**, and **3** gelators in sunflower oil, we did not notice a direct relationship to a more pronounced sustained release property of gelator **2**. Nevertheless, it should be considered a possibility of gelator–drug (AS) no-covalent interactions, especially in the case of polar terminal groups present in **2** and **3** gelator structures. Different viscoelasticity parameters have been observed during rheological measurements, giving into account the possibility of existence of dissolved gelator **2** molecules present in an equilibrium with the self-assembled aggregates in gelled oil (gel strength and loss factor of **2** lower compared to **1**, **3** gels). 

**Figure 13:**
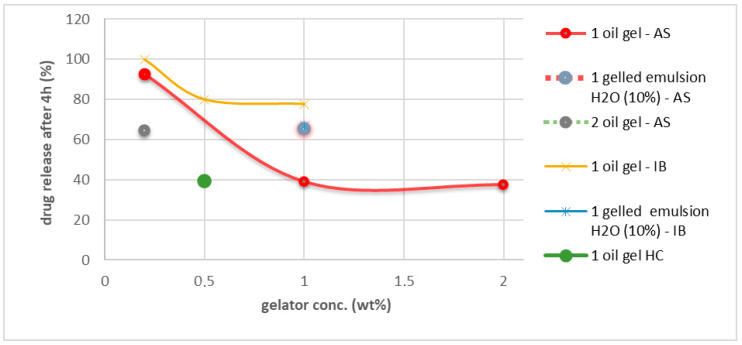
Controlled released profile of acetylsalicylic acid (AS) determined after 4 h from **1** sunflower oil gel of different concentrations to water phase (0.2 wt%; 1 wt%; 2 wt%; red curve), **1** oleogelled w/o emulsion (10% H_2_O (*v*/*v*) 1 wt%; blue dot), **2** sunflower oil gel (0.2 wt%; grey dot); a controlled released profile of ibuprofen (IB) from **1** sunflower oil gel of different concentrations (0.2 wt%; 0.5 wt%; 1 wt%; yellow curve) and a controlled released profile of hydrocortisone (HC) from **1** sunflower oil gel (0.5 wt%, green dot) to ethanol phase.

Additionally, we have demonstrated the possibility to entrap different drugs in oil gels, such as ibuprofen and hydrocortisone. As ibuprofen and hydrocortisone have low solubility in water, which we proved with poor released profiles of specified drugs in the water phase, the released experiments were performed as gels overlaid with ethanol. The 0.5 wt% of **1** oil gel showed release of 80% of IB in the first 4 h (Figure 13). The maximum retained capacity of the oleogel **1** (plateau) was reached at ~ 0.5 wt%, since the 1 wt% of **1** oil gel released 77.7% of IB in 4 h. If we compare the results for drugs of different polarity (1 wt% gel, release 40% AS and 0.5 wt% gel, release 80% IB), one can observe a threshold as a maximum capacity of the specified controlled delivery gels with different drugs released to different receptor media. 

Interestingly, the same gelator **1** of 0.5 wt% released only 40% of hydrocortisone (HC) in an ethanol layer in the first 4 h (Figure S5). The 0.5 wt% of **1** oil gel released ~70% of AS in the first 4 h of monitoring. The same concentration of gel **1** in sunflower oil (0.5 wt%) was prepared in all examined experiments. The proposed results of **1** oleogels (release of 40%-70%-80%) obtained for the different drugs (HC, AS, IB) of different polarity, diffusivity, and solubility in water/ethanol receptor media evidenced the strong influence of multiple parameters on controlled delivery experiments (Figure S6). The LC–MS analysis was performed under the same experimental conditions, resulting in different retention times of 7.4 min, 10.5 min, and 12.3 min for AS, HC, and IB, respectively. Since ibuprofen among other drugs showed the highest lipophilicity, one could expect a slower release profile of IB compared to HC (since released properties of IB and HC were monitored in ethanol). Comparing all the parameters, it can be assumed that the solubility of the specified drug from the same gel network had the most prominent impact on sustained release properties. The solubilities of IB and HC in ethanol are 60 mg/mL and 25 mg/mL, respectively. In the first 4 h of monitoring, the determined amounts were 80% and 40% of IB and HC, respectively. The most polar compound, AS, showed also fast release properties (70%), but direct correlation was not possible since the release of AS was monitored in water. The **1** oleogelled w/o emulsion (10% H_2_O, 1 wt%) encapsulating IB showed a slower release profile compared to pure **1** oil gel; in the first 4 h, they released 67% of IB and 77.7% of IB, respectively. These results confirm the dependence of IB low solubility in the water phase and consequently slower diffusivity from emulgel to receptor media (Figure S5). 

We have shown the versatile possibilities entrapping drugs of different polarity and solubility in the oil gels of different gelator structures and concentration. The examined oil gels offer a wide range of possibilities for the delivery of pharmaceuticals mainly for topical purposes. Nevertheless, the **1** gels overlaid with water of pH = 2 showed stability for days, offering the potential for oral drug delivery systems with a possible protection of the gastrointestinal surface in stomach. The examined gels with potent self-healing properties could also be used as delivery systems in injections. Nevertheless, the application of different drug/bioactive/nutraceutics delivery aspects could be envisaged in pharma and food, and application in cosmetics could also be foreseen. 

### 2.7. Fat Substitute Experiments Applied on Food Products

The use of organogels as a solution to replace and reduce trans and saturated fats has led to great advances in the recent years [61,62]. Considering the role of saturated fats in controlling the structure, texture, and taste of different products, their replacement represents a great challenge to the food industry. The organogelator is capable of self-assembling in nanoaggregates, forming a 3D network in a complex media only through no-covalent interactions. These interactions are affected by external stimuli such as temperature, shear, solvent used, which in turn strongly affect the gel network properties. Patel at al. made a comparative evaluation of structured oil systems based on the oleogel structuration self-assembling principles [63]. Fat mimetics as oleogels can produce a solid-like texture in food products with an improved level of unsaturated fatty acids present in liquid oil. In addition to the improvement in the nutritional profile of oleogels, the investigation showed that oil microencapsulation attenuates the acute elevation of blood lipids and insulin in humans [64]. As delivery vehicles, they showed a controlled rate of release of bioactive compounds in foods [65].

The ability of organogels to structure oil can also prevent oil migration in cream-filled cookies, which is usually seen as a texture defect. The use of ethylcellulose to form a stable heat-resistance system combined in chocolate was demonstrated up to 70 °C [66]. This effect was found to be more significant in milk and white chocolate than in dark chocolate, implying the involvement of the sugar molecules through hydrogen bondings with ethylcellulose in this process [67]. The main stability issue in confectionary products is the migration and leakage of oil under storage conditions. Patel et al. studied the behaviour of shellac oleogels in chocolate pastes (replacing 27% of the palm oil) as efficient oil binders [68]. Confectionary products stabilized with the use of 1–2 wt% of shellac wax or cellulose derivatives showed comparable stability and rheological properties to the reference samples. The World Health Organization has given instructions to the food industry to cut down the amount of solid fats or palm fats in manufactured food products. Solid fats are responsible for the solid state, texture, spreadability, and taste of millions of products. It is not easy to find an efficient substitution for solid fats regarding high demands in different areas. There is a huge need in the food sector to find a rational solution in the form of functional fat substitutes with positive health implications that is easy-to-use and has economic viability. One of the biggest disadvantages of oleogelators is their low gelation efficiency. Artificially designed organogelators showed potent gelation efficacy, representing powerful unidirectional self-assembly driven by no-covalent interactions.

We have preliminary investigated the possibility of the substitution of the total amount of solid fats in a complex sweet spread matrix with sunflower oil and organogelator **1** (0.03–0.2 wt%). The preparation procedure of milk and cocoa spreads were described in the section Materials and Methods. The samples of various spreads were prepared using the same ingredients with and without the addition of palm fats (7% total amount). An amount of 7% of palm fats contained in the reference samples is responsible for the solid state, texture, and spreadability of the prepared products. We decided to substitute 7% of palm fats with 7% of sunflower oil and specified concentration of gelator **1**. All other ingredients were added in the same amounts and followed the beforementioned preparation procedure. To examine the flowing character of the test samples with added sunflower oil, but without the presence of gelator molecules, the test tube inversion experiments and viscoelastic properties were obtained. The prepared mixtures of gelator **1** and sunflower oil showed excellent stability for a prolonged period of time (in months, Figure S7). The gelled samples were prepared at different gelator concentrations to demonstrate the influence of the new structuring agent on viscoelastic properties of potentially new food products without the addition of palm fats. 

#### Rheology of Fat Substitutes in Food Spreads

**Figure 14:**
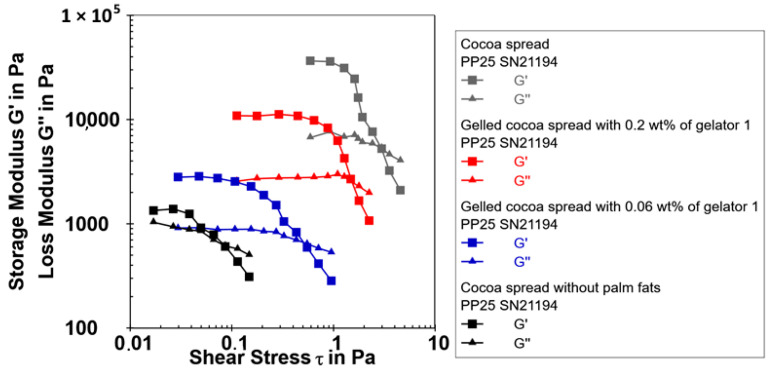
Amplitude sweep tests (G′ and G″ values) of cocoa spread (with added palm fats), cocoa spread without palm fats (with sunflower oil instead), gelled cocoa spread with 0.06 wt% gelator **1**, and gelled cocoa spread with 0.2 wt% gelator **1** at angular frequency 10 rad s^−1^ and 23 °C.

In this study, the viscoelastic properties of the investigated samples were determined using amplitude oscillatory tests in the range of 0.01–100% shear strain and constant frequency of 10 rad s^−1^. Figure 14 represents the amplitude sweep of different variations of cocoa spreads, including gelled samples with sunflower oil and different concentrations of gelator **1** and test sample with added sunflower oil instead of the total amount of solid fats in comparison to the reference cocoa sample containing palm fats. The cocoa spread with added palm fats as a structuring agent used as the reference sample showed the highest values of examined viscoelastic parameters (Table 7). The highest strength (G′ 36,787 Pa), yield point (1.5 Pa), and the lowest loss factor (tan δ = 0.18) were determined for the cocoa spread with added palm fats used as the reference sample. The sample of cocoa spread without added palm fats, but with addition of liquid sunflower oil instead of palm fats showed 25-times lower storage modulus (G′ 1388 Pa) and high relative elasticity (tan δ = 0.68); consequently, the investigated sample possesses higher G′ > G″, but the gravity immediately causes the flowing characteristics of the tested sample (Figure S7).

The viscoelastic properties of the newly prepared test samples of cocoa spreads with the addition of sunflower oil and gelator **1** mirrored the power of self-assembly at the nano level at exceptionally low concentrations of the gelator molecules. The gelled cocoa spread with only 0.06 wt% gelator **1** showed a storage modulus G′ of 2859 Pa and a relative elasticity (tan δ) 0.32. An additional increase in the concentration of gelator **1** influences the viscoelastic parameters comparable with the reference sample containing palm fats. The viscoelastic properties of the 0.2 wt% gelled cocoa spread were G′ 11,245 Pa, yield point 0.8 Pa, and relative elasticity 0.25 (Figure 14). It is very interesting to note that a higher concentration of gelator **1** caused four-times higher storage moduli (G′) and an additional decrease of the loss factor. The relative elasticity about 0.2 represents an important factor for the spreadability of food products. It can be assumed that with an additional amount of gelator **1,** one would be able to closely mimic the viscoelastic properties of the reference sample. 

**Figure 15:**
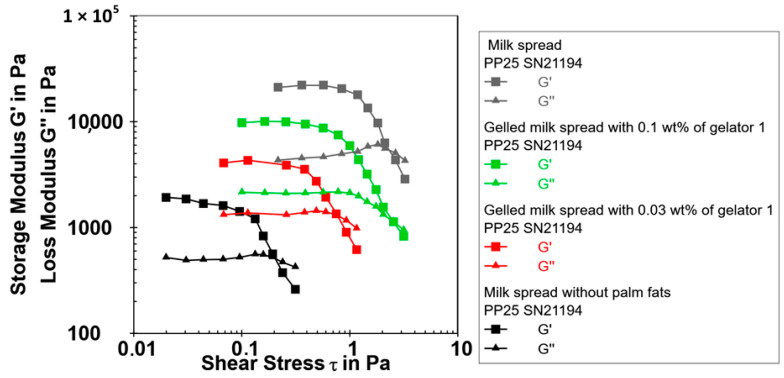
Amplitude sweep tests (G′ and G″ values) of milk spread (with palm fats), milk spread without palm fats (with sunflower oil instead), gelled milk spread with 0.03 wt% gelator **1**, and gelled milk spread with 0.1 wt% gelator **1** at angular frequency 10 rad s^−1^ at 23 °C.

Figure 15 represents the amplitude sweep of different variations in milk spreads, including gelled samples with sunflower oil and different concentrations of gelator **1** and test sample with added sunflower oil instead of the total amount of solid fats in comparison to the reference milk sample containing palm fats. 

The milk spread with added palm fats showed lower strength compared to the corresponding cocoa spread sample. The storage modulus G′ of the milk spread used as a reference sample was 22,253 Pa, yield point 1.1 Pa, and loss factor tan δ = 0.20 (Table 8). The milk spread containing sunflower oil instead of palm fats showed much a lower value of G′ 1687 Pa and loss factor of 0.29. It is interesting to observe that the milk spread with added sunflower oil had higher structural organisation compared to the corresponding cocoa spread (tan δ 0.68). The mixture of sunflower oil with gelator **1** at exceptionally low concentration 0.03 wt% gave a positive impact on the final composition (G′ 4082 Pa, yield point 0.4 Pa). The gelled milk spread with 0.1 wt% of gelator **1** showed a further increase of oleogelled mixtures strength (G′ 9994 Pa, yield point 0.8 Pa) and an impressive relative elasticity (tan δ) 0.21.

The substitution of palm fats with sunflower oil and 0.1 wt% of gelator **1** showed almost similar viscoelastic properties to the milk spread with added palm fats used as a reference sample, with somewhat weaker strength but still closely mimicking most of the other viscoelastic properties. The viscoelastic properties of the reference milk spread sample could be mimicking at a lower gelator concentration (0.1 wt%) compared to the reference cocoa spread sample. The presented results clarify the influence of a formulation matrix on the synergistic interactions among the tested gelator and milk spread ingredients, capable of forming additional interactions contributing to more efficient structuring of the final composition at much lower gelator concentrations. It is interesting that a small amount of gelator molecules (0.03–0.2 wt%) showed a tendency to form self-assembled network of aggregates in a complex media such as cocoa and milk spread constituted of different solid ingredients. 

However, the question is do we have to completely mimic the viscoelasticity of the original products structured with solid fat, or the newly prepared samples could be potentially acceptable in a wider range of viscoelastic properties since solid fats form microstructured and organogels a nanostructured network with enhanced physical properties. Nevertheless, a huge amount of investment is needed in the case of a new additive regulatory approval. Even though microstructural and rheological properties of fat substitutes, such as organogels, are different from fats, when they are used to replace fat in food products, the end products do show comparable properties to a standard formulation. 

As delivery vehicles, fat mimetics as organogels can offer multiple advantages such as providing stabilization against crystallization, preventing oxidation of bioactives, and controlling a rate of release of various bioactives. The ability of oleogels to form a stable heat-resistance system, preventing oil migration in cream fillings and cookies and helping to prolong the shelf-life provides further investigation into different low molecular weight organic gelators as promising fat substitutes. 

## 3. Conclusions

The current research aimed to expand the knowledge on oxalamide derivatives as powerful oil gelling systems. The gelation behaviour of chiral oxalamide derivatives was investigated in different vegetable oils. The compounds classified as supergelators (**1**, **2,** and **3**) with a minimal gelator concentration of 0.026 wt% to 0.1 wt% and self-healing properties were investigated. Prepared oil organogels revealed important features such as thermoreversibility and thixotropy. This represents an important exploration concerning the development of oil-based soft matter systems for food structuring applications and the controlled delivery of bioactives. The examined oxalamide compounds have been previously described as weak or moderate gelators of organic solvents, solvent mixtures, and mixtures with water. Interestingly, gelators **1**–**3** showed exceptional gelling ability in different vegetable oils with very potent thixotropic properties never mentioned in the previous examination. The gels prepared in different oils were transparent gels stable for years, which endows a number of potential applications. The chiral oxalamide gelators showed the existence of self-assembled aggregates of 40–400 nm width in sunflower oil. The self-healing properties of the oil gels determined in 3ITT consecutive cycles represent proof of self-recoverable viscoelasticity of distinguished gelators as a potential solution for specific applications in food, cosmetics, and pharma. 

Additionally, gelator **1**, an ester derivative, is capable of gelling oil in the presence of a small amount of water (10–20% H_2_O, *v*/*v*), forming translucent or opaque gelled emulsions stable for months. Even though all investigated oxalamide gelators showed exceptional gelation in various oils, only gelator **1** could cause gelation of w/o emulsions. This particular information is very important from different aspects. The oxalamide derivatives **2** and **3** are chiral oxalamide gelators with terminal acid and amide groups, respectively. An addition of water in **2** oil gels cause initial precipitation of the compound **2** and formation of bulky aggregates of **3**. Similar remarks of instabilities related with water presence in **3** oil gels were noticed during sustained drug delivery experiments. The gelator structure, the balance of lipophilic and hydrophilic groups, their spatial arrangement, the number of hydrogen bonding units, and presence of different functional groups capable of forming additional no-covalent interactions represents the key in the creation of smart soft functional materials. Addition of water in **1** oil gel causes the formation of broader twisted ribbons with widths of 100 nm–1 μm in **1** gelled w/o emulsion and consequently higher gel enthalpy changes compared to **1** oil gel. These observations could be explained by the unfavourable aggregate–solvent interactions (aggregate solvation), which in the case of polar solvents, induces the formation of wide ribbons to decrease the lipophilic surface of the aggregates exposed to polar solvents. The **1** gelled w/o emulsion showed somewhat lower elastic moduli, similar relative elasticity but a twofold lower yield point value and poor self-recovery was noticed after mechanical agitation compared to pure **1** oil gel. 

Regarding the excellent gelation ability of the presented gelators in various oils, it seems they possess an optimum balance between the affinity and insolubility in the oil phase. The viscoelastic parameters of sunflower oil gels decreased in the subsequent order: **1** > **3** > **2**. The highest elastic moduli, yield point, and flow point values in sunflower oil were determined for gelator **1**. The **3** oil gel showed the lowest relative elasticity of 0.16. The recovery of G′ and G″ values in two consecutive thixotropic recovery cycles was determined. Similar self-recovery in sunflower oil was observed for gelators **1** (80.1%) and **3** (84.8%), lower in **2** (50%), and poor recovery of the **1** gelled w/o emulsion (10% H_2_O; 37%). 

The influence of the oil type (sunflower, soybean, and olive oil) on gelation capacity and viscoelastic properties have been examined. The interplay between gelator–gelator and gelator–solvent interactions deeply depend on the chemical composition and the polarity of the oil. Using different oil types for structure formation by oxalamide **1** gelator, G′ decreased as sunflower oil > soybean oil > olive oil indicated a higher amount of gelator–solvent interactions in a more polar environment, causing weaker gel strength. Regarding gelation properties of gelator **1**, it could be concluded that the gelation efficacy is similar in sunflower oil and soybean oil, but more pronounced in olive oil (more than 30%). Contrary to the pronounced gelation efficacy noticed in olive oil, the self-healing properties were more pronounced in sunflower and soybean oil. This provides the evidence of the impact of the oil composition, a ratio of MUFA/PUFA acids, and presence of small percentage of various polar components capable of interacting with gelator molecules. Depending on the gelator structure, the synergistic effect of oil composition clearly influenced gelation capacity in certain cases. Nevertheless, the self-healing phenomena observed after mechanical disruption of the gel network is also influenced by the oil polarity since gelator–solvent interactions play a key role in re-establishment of broken fibre aggregates and 3D network capable of entrapping oil and preventing it from flowing. We assume the polar components in olive oil (1–2%), amongst other factors influence higher gelator–solvent interactions and consequently cause weaker gel strength and slower recovery, as is observed in olive oil gels. 

After detailed study presented in the paper, we could assume the powerful influence of the gelator structure, self-assembly of gelator molecules at nano scale, entanglement of aggregates at microscale, 3D gel morphology capable of entrapping oil to a high extent, influence of the oil polarity, viscosity, chemical composition of oil phase, spatial arrangement of hydrocarbon chains in fatty acid composition, oil–gelator interactions, and optionally interactions with various bioactive molecules present in different compositions in various oils affecting altogether in a complex manner the gelation potency and self-recovery of the investigated oil gelators. Certainly, the influence of gelator structure in a wide range of oils should be investigated in detail. Additionally, we have examined the versatile possibilities of entrapping drugs (acetylsalicylic acid, ibuprofen, and hydrocortisone) of different polarity and solubility in the oil gels of different gelator structure and concentration. If we compare the sustained release properties of AS (64–100%) of the three structurally different oxalamide gelators (**1**, **2**, and **3**) examined at the same concentration (0.2 wt%) in sunflower oil, one can observe a deep influence of the gelator structure, consequently self-assembly at nanoscale, gel morphology, maximum gelling capacity, and an influence of viscoelasticity of the 3D gel network at macroscale. Addition of water in **1** oil gel causes the formation of different gel network morphology, and water droplets in **1** gelled w/o emulsion support faster diffusion rate of the entrapped drug (AS) to the surface water layer. Monitoring of the concentration dependence released profiles of different drugs enabled determination of the maximum capacity of investigated **1** oil gel network to different receptor media (~1 wt% for AS, and ~0.5 wt% for IB). The proposed results of **1** oleogels (release of 40%, 70%, and 80%) obtained for the different drugs (HC, AS, IB, respectively) of different polarity, diffusivity, and solubility in water/ethanol receptor media evidenced the strong influence of multiple parameters on controlled delivery experiments. The examined oil gels offer a wide range of possibilities for the delivery of pharmaceuticals as controlled delivery systems. 

To explore the versatile potential of oxalamide derivatives, gelator **1** was used as a fat substitute in sunflower oil instead of solid fats. Fat substitutes as oleogels can produce a solid-like texture in food products with an improved level of unsaturated fatty acids present in liquid oil. The experiments of solid fat substitution with gelator **1** combined with sunflower oil were performed in cocoa and milk spreads. The results of the promising substitution compared to the reference samples were evident even at 0.1 wt% of gelator **1** in milk spread samples and 0.2 wt% in cocoa spread samples. The presented results clarify the influence of a formulation matrix on the synergistic interactions among the tested gelator and milk spread ingredients, capable of forming additional interactions contributing to a more efficient structuring of the final composition at much lower gelator concentrations. The relative elasticity, a very important structural parameter, value of ~0.20 of the tested gelled spreads was reached. We demonstrated the influence of the new structuring agent as a potent fat substitute on the viscoelastic properties of different spreads at considerably lower gelator concentration compared to the published results. The ability of oleogels to form a stable heat-resistance system, providing stabilization against crystallization, preventing oxidation of bioactives, controlling a rate of release of various bioactives, preventing oil migration in cream fillings, and helping to prolong shelf-life provides further investigation into different low molecular weight organic gelators as promising fat substitutes. 

The examined oxalamide oil gels showed advantageous properties, such as thermoreversibility, high temperatures of gel–sol transition as a potential heat-resistance material, suitability of viscoelastic properties, self-healing properties, potential in sustained delivery of different drugs, and gelation efficiency at exceptionally low concentrations in various oils and complex food systems. Although the oxalamide derivatives are not food grade structuring agents, their gelation potential in oils should be examined from a fundamental point of view to highlight the importance of specific structural features in searching for the most favourable candidates with desirable properties. A careful examination of various classes of organogelators offers new insights into the important aspects of the self-assembling nature in various oils, resulting in attractive and outmost applicable properties. 

## 4. Materials and Methods

The synthesis of the compound **1** has been prepared according to the procedure described in the paper by Šijaković et al. [43]. The compounds **2** and **3** have been prepared and characterised according to the paper by Makarević et al. [12]. The synthetic procedure is described in the Supplementary Material. Three different oils were used in the current study. Sunflower oil and extra virgin olive oil were purchased at a local supermarket and used without purification. Soybean oil was purchased from Alfa Aesar. 

### 4.1. Determination of Gelling Properties

All gelation experiments were performed in test tubes of 12 mm in diameter. The tested substance was placed in a test tube, and the oil was added in 500 µL portions. After each addition, the mixture was gently heated until the substance dissolved and was then allowed to cool spontaneously to room temperature. The formation of a gel is checked by test tube inversion. The heating–cooling procedure is repeated until the formation of a loose gel or dissolution is observed. Gelation properties of the compounds were tested against various edible oils and the results are collected in Table 1 and Table S1. The gelation efficiency of each gelator toward the specified edible oil is expressed in mL of oil that could be immobilized by 10 mg of the gelator. All of the prepared gels in different oils were transparent and showed thermoreversible gel–sol transitions.

### 4.2. Determination of Thixotropic Property

The gels were subjected to external mechanical stress (shaking) till the gel was transformed into a liquid state. The self-healing process (or recovery to a gel state) after standing at room temperature was examined every 5 min by test tube inversion method and visual observation. The time of response necessary to self-recover from sol to gel state was determined at a half of the maximum gelling volume per 10 mg of the tested compound (Table 1, column Recovery time (min)).

### 4.3. Determination of Gel Melting Temperatures

The gel melting temperatures were determined with the dropping ball set-up measurements. Oil gels were made in test tubes 1 day before testing. A stainless-steel ball with a diameter of 2.5 mm (90.32 mg) was placed on the top of gels, and the gels were slowly heated in the stirred oil bath. The oil gels were considered melted when the ball had reached the bottom of the test tube. The oil gelled emulsions were considered melted when the aggregates were dissolved and transparency of the system was obtained. The dropping ball experiments were carried out twice, and the melting temperatures obtained were reproducible to within 1 °C.

### 4.4. TEM Investigation

The specimens were examined in a Zeiss EM 10A transmission electron microscope operating at 60 kV. For electron microscopy, a piece of gel was placed on a copper grid and removed after 20 s, leaving some patches of the gel on the grid. Morphologies of self-assembled fibres of different gelators were negatively stained by dipotassium polytungstate and Pd shadowing. 

### 4.5. Differential Scanning Calorimetry

DSC was carried out on a Perkin-Elmer DSC 7. For measurements on the gels, a weighed amount of gelator together with a weighed amount of oil was placed in a 60 µL stainless steel cup which was sealed directly. The sample cup was placed in the DSC apparatus together with an empty sample cup as a reference. Heating and cooling scans of the oil gels were recorded at a scan rate of 5 °C min^−1^. Repeated heating and cooling scans of the gel samples were reproducible, and prolonged aging times did not affect the results. 

### 4.6. Rheological Investigation

The mechanical properties of the gels are described using oscillatory rheology measurements. The storage (G′) and loss (G″) moduli of the gels were determined with a mechanical rheometer (Anton Paar MCR 302, Stuttgart, Germany), using a steel plate−plate geometry (PP25, rubbed surface) equipped with a true-gap system, and the data were collected using RheoCompass software. The temperature is controlled through a Peltier temperature control located on the base of the geometry and with a Peltier-controlled hood (H-PTD 200). A slice of a gel sample (1 mm thick slice) was placed on the base plate of the rheometer, and the plate was set using the true-gap function of the Rheo software. After 15 min at 15 °C, the G′ and G″ moduli were measured within the linear viscoelastic region (LVR). The yield stress of the oil gels was determined by applying a strain (γ) sweep between 0.01% and 100%. Rheological properties of the gels were independent of the strain up to yield strain, and beyond yield strain, the rheological behaviour is nonlinear. Three interval thixotropy test (3ITT) allows the tracking of response resulting from stepwise changes in shear strain, making it the most appropriate method for structure recovery tests. In 3ITT tests, rheological measurements were conducted on gels at 15 °C under initial conditions at which they were in their linear viscoelastic regimes (a strain of 0.1% and angular frequency of 10 rad/s) for 500 s to establish baseline values for G′ and G″ moduli. The viscoelastic recovery of the gels was observed after the cessation of a destructive strain. Frequency sweeps (0.01–100 rad/s) were performed at 15 °C at a strain value within LVR to investigate the time-dependent deformation behaviour of oil gels.

### 4.7. Evaluation of Drug Release from Oil Gel

To investigate a release profile of the selected analytes from prepared gels, the simple LC–MS method for determination of acetylsalicylic acid, ibuprofen, and hydrocortisone was developed.

#### 4.7.1. Chemicals

Acetylsalicylic acid for the standard preparation was purchased from TCI (Zwijndrecht, Belgium) with a purity ˃ 98.0%. Ibuprofen and hydrocortisone as the pharmaceutical reference materials were obtained from Merck. Ethanol absolute was purchased from Riedel-de Haën (Seelze, Germany). MiliQ^®^ water (18.2 MΩcm^−1^; purified by MiliQ water purification system (Millipore, Bedford, MA, USA)) and HPLC gradient-grade methanol (J.T.Baker, Center Valley, PA, USA) were used with analytical-grade formic acid (FA) (Acros Organics, Geel, Belgium) for the mobile phase preparation. Stock solutions of acetylsalicylic acid, ibuprofen, and hydrocortisone were prepared as 1 mg/mL solutions in methanol. 

#### 4.7.2. Sample Preparation and LC–MS Condition

The gels with approximately 2 mg of the specified drug were prepared in a well-closed glass vial and stored at room temperature. The gel surface was overlaid with 2 mL of MiliQ water or ethanol. The released properties of acetylsalicylic acid were monitored in the water phase and ibuprofen and hydrocortisone in ethanol. Every selected time point after addition of water/ethanol on the gel surface, including immediately after the addition, two 50 μL sample aliquots were sampled for determination of the release rate of the investigated drug from the prepared gels. The sampled aliquots were replaced with an equal volume of fresh of MiliQ water/ethanol to maintain the total volume of the solution over the gel. In one aliquot, a corresponding volume of stock solution was added (spiked sample) and in the second, the same volume of methanol was added (non-spiked sample). A total of 5 µL of clear solution was injected on the LC column. During the analysis, all instrumental blank samples were negative. Quantification of the analyte was performed by monitoring the difference in the peak area of the investigated drug in the non-spiked sample and the spiked sample prepared with the addition of the known concentration of the standard.

LC–MS analysis was carried out using an Agilent Technologies 1200 series HPLC system equipped with a binary pump, a vacuum membrane degasser, an automated autosampler and injector interfaced with 6420 triple quadropole mass spectrometer with electrospray ionization source (ESI) (Agilent Technologies Inc., Palo Alto, CA, USA).

The analysis was performed on Zorbax XDP C18 column (75 × 4.6 mm, 3.5 μm particle size) (Agilent Technologies Inc., Palo Alto, CA, USA). Solvents for the analysis were 0.1% formic acid (FA) in water (solvent A) and methanol (solvent B). The gradient was applied as follows: 0 min 70% A, 0–10 min 70% A–0% A, 10–12 min 0% A, 12.1–15 min 70% A. Flow rate was 0.5 mL/min 

The electrospray ionization source was operated in a positive mode and samples were detected in the single ion monitoring (SIM) mode. Acetylsalicylic acid was monitored at *m*/*z* 203 as a [M + Na]^+^ adduct. Ibuprofen was monitored at *m*/*z* 229 as a [M + Na]^+^ adduct in the positive ionization SIM mode. Hydrocortisone was monitored at *m*/*z* 363 as [M + H]^+^ ion in the positive ionization SIM mode. The fragmentor voltage was set at 100 V. The desolvation gas temperature was 300 °C with a gas flow rate of 8.0 L/min The capillary voltage was 4.0 kV. The retention times of acetylsalicylic acid, ibuprofen, and hydrocortisone were 7.4 min, 12.3 min and 10.5 min, respectively. All data acquisition and processing were performed using Agilent MassHunter software.

### 4.8. Preparation Procedure of Various Food Spreads 

For the purposes of this research, food spreads were laboratory samples of cocoa and milk spreads (in text as cocoa and milk spreads), prepared by the food industry. All ingredients, with or without palm fats (basic base), were mixed together and then transferred on the laboratory ball mill (Duyvis Wiener type W-1-S) on a conching process for obtaining a spread.

#### 4.8.1. Cocoa Spread Sample

Ingredients (the basic base): sugar, fats (quantity and variations later described), skimmed milk powder, fat-reduced cocoa powder, whey powder, dextrose, soy lecithin.

Samples (basic base) had different fat origin for the investigation.

Reference spread was with 7% of added palm fats (a), fully replaced palm fats with the equivalent amount of sunflower oil (b), and fully replaced palm fats with sunflower oil and specified amount of gelator **1** (0.06 wt%, 0.2 wt%) (c).

#### 4.8.2. Milk Spread Sample

Ingredients (basic base): sugar, fats (quantities and variations later described), whole milk powder, whey powder, soy lecithin.

Samples (basic base) had different fat origin for the investigation.

Reference spread was with 7% of added palm fats (a), fully replaced palm fats with equivalent amount of sunflower oil (b), and fully replaced palm fats with sunflower oil and specified amount of gelator **1** (0.03 wt%, 0.1 wt%) (c).

#### 4.8.3. Preparation of Gelled Spreads

The specified amount of gelator **1** (wt%) was dissolved in 4% of sunflower oil (total amount of initial fats in a final composition is 7%). 

All other beforementioned ingredients plus 3% of sunflower oil were mixed together and transferred to a laboratory ball mill (Duyvis Wiener type W-1-S) on conching process for obtaining a spread. A previously prepared hot solution of oil component with gelator **1** was manually added to the prepared spread, vigorously mixed (1–2 min) and left at room temperature for 10 min. The samples were checked for their flowing properties by the test tube inversion method.

## 5. Patents

Vujičić, N. Š. Low molecular weight organic gelators of vegetable oil. CA Patent No. CA3022218C, 9 May 2023.

## Figures and Tables

**Table 1:** Gelation efficiency of the compounds **1**, **2**, and **3** toward the specified edible oil expressed as the maximal volume (Vmax/mL) of oil that could be immobilized by 10 mg of the gelator. Thixotropic property measured at half of the maximum gelling volume by visual observation of the test tube inversion method.

Compound	Sunflower Oil V/mL	Recovery Time */min	Soybean Oil V/mL	Recovery Time/min	Olive Oil V/mL	Recovery Time/min
**1**	17.3	5 min	16.6	5 min	25.9	30 min
**2**	13.2	5 min	43.3	5 min	27.2	10 min
**3**	9.4	5 min	14.5	5 min	15.2	5 min

* Self-healing properties of gels were checked by test tube inversion method every 5 min.

**Table 2:** ΔH_m_, T_m_, and T_c_ values obtained from DSC measurements of **1** sunflower oil gel and gelled emulsions (10% and 20% H_2_O, *v*/*v*) at concentration of 2.5 wt%.

Sample	T_m_ (°C)	ΔH_m_ (kJmol^−1^)	T_c_ (°C)
1 sun	141.6	17.86	116.7
1 sun/water (10%)	131.1	36.85	98.8
1 sun/water (20%)	142.8	46.03	115.0

**Table 3:** Results of amplitude sweep tests of 0.2 wt% of **1**, **2,** and **3** sunflower oil gels and **1** oil gelled w/o emulsion (10% H_2_O, *v*/*v*; **1** sun/water) examined at 15 °C.

				Yield Point/Pa		Flow Point/Pa	
Sample	G′/Pa	G″/Pa	tanδ	τ/Pa	γ/%	τ/Pa	γ/%
1 sun	1371.1	264.3	0.19	22.2	2.2	48.4	9.3
3 sun	1102.1	176.2	0.16	12.4	1.7	22.7	6.8
1 sun/water	1031.1	202.4	0.20	12.6	1.6	28.7	9.6
2 sun	708.9	189.9	0.27	1.2	0.2	5.7	3.2

**Table 4:** Self-healing properties of the 0.2 wt% of **1**, **2,** and **3** sunflower oil gels and **1** gelled w/o emulsion (10% H_2_O; **1** sun/water) determined in 3-interval thixotropy test at 15 °C.

1.–3. Interval			
Sample	Recovery (*t* = 60 s)/%	Recovery (*t* = 300 s)/%	Recovery (*t* = 600 s)/%
1 sun	67.2	75.5	80.1
3 sun	52.7	81.4	84.8
1 sun/water	31.6	35.1	37.1
2 sun	39.1	47.6	50.0

**Table 5:** Results of amplitude sweep test of 0.2 wt% of **1** sunflower oil gel (1 sun), **1** soybean oil gel (1 soy), and **1** olive oil gel (1 olive) performed at 10 rad s^−1^ and 15 °C.

				Yield Point/Pa		Flow Point/Pa		Yielding Zone
Sample	G′/Pa	G″/Pa	tanδ	τ/Pa	γ/%	τ/Pa	γ/%	Pa
1 sun	1371.8	264.3	0.19	22.2	2.2	48.4	9.3	26.2
1 soy	1042.1	208.6	0.20	17.0	2.3	39.6	10.9	22.6
1 olive	628.3	118.5	0.19	14.1	3.0	37.0	16.8	22.9

**Table 6:** Self-healing properties of 0.2 wt% of **1** sunflower oil gel (1 sun), **1** soybean oil gel (1 soy), and **1** olive oil gel (1 olive) determined in 3-interval thixotropy test.

3ITT			
Sample	Recovery (*t* = 60 s)/%	Recovery (*t* = 300 s)/%	Recovery (*t* = 900 s)/%
1 sun	80.1	91.0	95.0
1 soy	64.8	74.7	81.5
1 olive	58.9	68.5	74.8

**Table 7:** Results of the amplitude sweep tests of the various cocoa spread samples: with palm fats (referent sample), palm fats fully replaced by sunflower oil, and palm fats fully replaced by sunflower oil and various concentrations of gelator **1** (0.06 wt% and 0.2 wt%) determined at 23 °C.

				Yield Point/Pa		Flow Point/Pa	
Sample	G′/Pa	G″/Pa	tanδ	τ/Pa	γ/%	τ/Pa	γ/%
Cocoa spread (with palm fats)	36,787.0	6759.8	0.18	1.5	0.005	3.0	0.04
Cocoa spread without palm fats (with sunflower oil)	1388.3	938.58	0.68	0.04	0.003	0.08	0.01
Gelled cocoa spread with 0.06 wt% of gelator **1**	2859.9	916.26	0.32	0.2	0.009	0.5	0.06
Gelled cocoa spread with 0.2 wt% of gelator **1**	11,245.0	2763.5	0.25	0.8	0.009	1.5	0.04

**Table 8:** Results of the amplitude sweep tests of the various milk spread samples: with palm fats (referent sample), palm fats fully replaced by sunflower oil, and palm fats fully replaced by sunflower oil and various concentrations of gelator **1** (0.03 wt% and 0.1 wt%) determined at 23 °C.

				Yield Point/Pa		Flow Point/Pa	
Sample	G′/Pa	G″/Pa	tanδ	τ/Pa	γ/%	τ/Pa	γ/%
Milk spread with palm fats	22,253.0	4514.1	0.20	1.1	0.006	2.4	0.03
Milk spread without palm fats (with sunflower oil)	1887.4	496.78	0.29	0.1	0.009	0.2	0.03
Gelled milk spread with 0.03 wt% of gelator **1**	4082.8	1321.1	0.32	0.4	0.01	0.8	0.04
Gelled milk spread with 0.1 wt% of gelator **1**	9994.0	2104.4	0.21	0.8	0.01	2.5	0.2

## Data Availability

Not applicable.

## Supplementary Materials

The following supporting information can be downloaded at: https://www.mdpi.com/article/10.3390/gels9090699/s1, Figure S1. TEM image of (a,b) **2** sunflower oil gel (fibres and ribbons of 100–400 nm), (c) **3** sunflower oil gel (fibres and ribbons of 40–400 nm in diameter; bar 1 μm); Table S1. Gelation efficiency of the compounds **4–14** towaFrd the specified edible oil expressed as the maximal volume (V_max_/mL) of oil that could be immobilized by 10 mg of the gelator. Thixotropic property measured at a half of the maximum gelling volume; Figure S2. TEM images of (a) **1** DMSO/water gel, ribbons with apparent twist (mostly 80–200 and up to 600 nm widths), (b) **1** tetralin gel (Pd shadowing) fibres of 8–20 nm d values; Table S2. Gel–sol phase transition temperatures of **1** sunflower oil gels and **1** gelled emulsions at 10% and 20% of water content obtained by the dropping ball method; Figure S3. 3-interval thixotropy test (3ITT) (G′ and G″ values) of the 0.2 wt% of **1** sunflower oil gels and **1** gelled w/o emulsion (10% H_2_O). Linear viscoelastic region (LVR): strain = 0.1%, frequency = 10 rad/s; destructive region (DR): strain = 100%, frequency = 10 rad/s; recovery region: linear viscoelastic region (LVR): strain = 0.1%, frequency = 10 rad/s; Table S3. Self-healing properties of the 0.2 wt% of **1**, **2**, and **3** sunflower oil gels and **1** gelled w/o emulsion (10% H_2_O, **1** sun/water) determined in 2nd consecutive recovery cycle of 3-interval thixotropy test in the 3.–5. Interval; Figure S4. Controlled release profile of acetylsalicylic acid from 1 sunflower oil gels at different gelator concentrations (0.2 wt% black; 1 wt% green; 2 wt% blue); 1 gelled w/o emulsion (10% H_2_O; 1 wt% red) and 2 sunflower oil gel (0.2 wt% grey) from initial point to until 4 h at 25 °C; Figure S5. Controlled release profile of ibuprofen from 1 sunflower oil gels at different gelator concentrations (0.2 wt% black; 0.5 wt% red; 1 wt% green); 1 gelled w/o emulsion (10% H_2_O; 1 wt% blue); and sustained release of hydrocortisone of 1 sunflower oil gel (0.5 wt% yellow) from initial point to until 4 h at 25 °C; Figure S6. Controlled release profile of acetylsalicylic acid from 1 sunflower oil gels at different gelator concentrations (0.2 wt% black; 1 wt% green; 2 wt% blue); 1 gelled w/o emulsion (10% H_2_O; 1 wt% red); 2 sunflower oil gel (0.2 wt% grey); sustained release profile of ibuprofen from 1 sunflower oil gels at different gelator concentrations (0.2 wt% blue; 0.5 wt% violet; 1 wt% yellow); 1 gelled w/o emulsion (10% H_2_O; 1 wt% cyan); and sustained release of hydrocortisone of 1 sunflower oil gel (0.5 wt% brown) from initial point to until 4 h at 25 °C; Figure S7. The comparison of (a) gelled milk spread (0.1 wt% of gelator 1 and sunflower oil) and milk spread with sunflower oil, without palm fats (flowing), (b) gelled milk spread, (c) gelled cocoa spread (0.2 wt% of gelator 1 and sunflower oil) and cocoa spread with sunflower oil, without palm fats (flowing), (d) gelled cocoa spread, (e) application of gelled cocoa and milk spread on bread; Figure S8. Stability of 1 gelled w/o emulsions in sunflower oil (10% H_2_O, *v*/*v*) for concentrations of 1.44 mmol/L; 2.0 mmol/L; 4.3 mmol/L; 7.6 mmol/L and 12.0 mmol/L in ascending order from the left to the right; (a) after 1 day, (b) 1 week, (c) 2 weeks, (d) 3 weeks, (e) 1 month, and (f) 2 months.
