# Supplementary material for: Self-Healing Oxalamide Organogelators of Vegetable Oil
Source: Gels. 2023 Aug 29;9(9):699. doi: 10.3390/gels9090699 (PMC10528235; doi:10.3390/gels9090699)
Supplement: Supplementary file 1 [file gels-09-00699-s001.zip › gels-2539553-supplementary.pdf]

Self-healing Oxalamide Organogelators of Vegetable Oil

Nataša Šijaković Vujičić [1,*] Josipa Suć Sajko [2], Lidija Brkljačić [2], Petra Radošević [1], Ivanka Jerić [2] and Ivona Kurečić [2]

Supplementary Material

Design, gelation properties, thixotropy and stability

Compound 2: *N*,*N′*-Oxalyl-bis((*S*)-leucylamide)

Compound 3: *N*,*N′*-Oxalyl-bis((*S*)-ValOH)

Compound 4: *N*,*N′*-Oxalyl-bis((*S*)-leucine methyl ester)

Compound 5: *N*,*N′*-Oxalyl-bis((*S*)-valylamide)

Compound 6: *N*,*N′*-Oxalyl-bis((*S*)-phenylalanylamide)

Compound 7: *N*,*N′*-Oxalyl-bis((*S*)-PheOH)

Compound 8: *N*,*N′*-Oxalyl-bis[(*S*)-phenylalanine methyl ester)]

Compound 9: (±)-[N,N'-Oxalyl-bis(phenylglycylamide)]

Compound 10: *N*,*N′*-Oxalyl-bis((*R*)-PhgOH)

Compound 11: *N*,*N′*-Oxalyl-bis[(*R*)-phenylglycine methyl ester)]

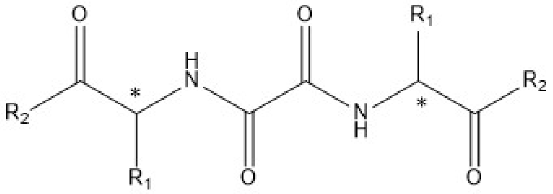

Compound	R¹	R²	Stereochemistry
2	$-CH_2CH(CH_3)_2$	$-NH_2$	*S,S*
3	$-CH(CH_3)_2$	-OH	*S,S*
4	$-CH_2CH(CH_3)_2$	$-OCH_3$	*S,S*
5	$-CH(CH_3)_2$	$-NH_2$	*S,S*
6	$-CH_2Ph$	$-NH_2$	*S,S*
7	$-CH_2Ph$	-OH	*S,S*
8	$-CH_2Ph$	$-OCH_3$	*S,S*
9	-Ph	$-NH_2$	*rac*
10	-Ph	-OH	*R,R*
11	-Ph	-OMe	*R,R*

Compounds **2-11** were synthesised according to the procedure described in Makarević at al., *Chem. Eur. J.* **2001**, *7* (15), 3328 – 3341.

Compound 1: 1,6-Bis((O-leucylmethanol)-*N*-yloxalamido)hexane

Compound 12: 1,6-Bis ((leucine)-*N*-yloxalamido)hexane

Compound 13: 1,9-Bis((O-leucylmethanol)-*N*-yloxalamido)nonane

Compound 14: 1,9-Bis ((leucine)-*N*-yloxalamido)nonane

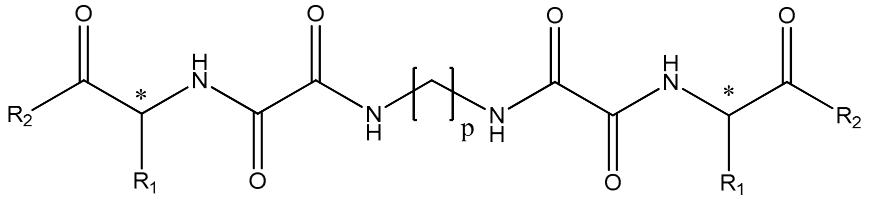

Compound	R¹	R²	p	Stereochem-istry
1	$-CH_2CH(CH_3)_2$	$-OCH_3$	6	*S,S*
12	$-CH_2CH(CH_3)_2$	-OH	6	*S,S*
13	$-CH_2CH(CH_3)_2$	$-OCH_3$	9	*S,S*
14	$-CH_2CH(CH_3)_2$	-OH	9	*S,S*

Compounds **1** and **12-14** were synthesised according to the procedure described in Šijaković Vujičić at al., *Chem. Eur. J.* 2013, *19*, 8558 – 8572.

EXPERIMENTAL

General

Melting points were determined on a Kofler stage. Optical rotations were measured on an optical activity AA-10 automatic polarimetar using the wavelength of 589.3 nm. The one- and two-dimensional homo- and heteronuclear ^{1}H and ^{13}C NMR spectra were recorded with a Varian XL-300 Gemini spectrometer, Bruker AV-300, and Bruker AV-600 spectrometers, operating at 300 and 600 MHz for the ^{1}H nucleus and 75 and 150 MHz for the ^{13}C nucleus, respectively. FTIR spectra were recorded on a Bomen MB 102 spectrometer. Thin layer chromatography (TLC) was performed on silica-gel-coated Merck 60 F_{254} silica plates and were visualized using a UV lamp (254 nm) or I_2 vapors. All chemicals were of the best commercially available grade and were used without purification. Solvents were purified according to methods described in the literature and stored over molecular sieves. Mass spectra were recorded on the Extrell FTMS 2001-DD Fourier Transform Mass Spectrometer electron impact, ionizing voltage 70 eV and 4800 MALDI TOF/TOF Analyzer, Applied Biosystems.

N, N'-oxalyl-bis(Aaa)-COOH

A solution of oxalyl chloride (1 mmol) in DCM (15 mL) and 4M aqueous KOH (4 mL) were simultaneously added dropwise to a cooled (- 10 °C) solution of the corresponding (*S*)-amino acid (2 mmol) in 4M KOH (6 mL). The stirring was continued for 30 min at 0 °C and for another 30 min at room temperature. Organic layer was separated and the aqueous layer diluted with water and acidified with 10 % citric acid. The gelatinous precipitate was left to stand at 4 °C for 2 h, filtered, and washed with water. The final product was obtained after recrystallization with MeOH/Et_2O as a white solid.

N, N'-oxalyl-bis(Val)-COOH

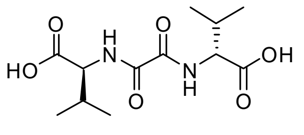

Chemical Formula: $C_{12}H_{20}N_2O_6$
Molecular Weight: 288,30

^{1}H NMR (300 MHz, DMSO) δ / ppm 8.10 (d, *J* = 8.6 Hz, 1H), 3.89 (dd, *J* = 8.5 Hz, 1H), 2.05 - 1.90 (m, 1H), 0.76 – 0.61 (m, 6H).

^{13}C NMR (75 MHz, DMSO) δ / ppm 172.4, 159.8, 58.5, 30.3, 19.6, 18.5.

N, N'-oxalyl-bis(Leu)-COOH

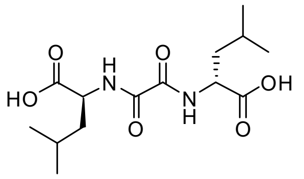

Chemical Formula: $C_{14}H_{24}N_2O_6$
Molecular Weight: 316,35

^{1}H NMR (300 MHz, DMSO) δ / ppm 8.45 (s, 1H), 4.08 (s, 1H), 1.58 (s, 3H), 0.86 (s, 6H).

^{13}C NMR (75 MHz, DMSO) δ / ppm 173.5, 159.7, 52.2, 25.0, 23.5, 22.2.

N, N'-oxalyl-bis(Aaa)-NH₂

To a *N*, *N′*-oxalyl-bis(Aaa)-COOH (1 mmol) dissolved in dry dichloromethane (DCM) (10 mL), *N*-methyl-morpholine (NMM) (2 mmol) was added and the solution was cooled down to 0 °C. Isobutyl chloroformate ($ClCO_2iBu$) (2 mmol) was added dropwise and the reaction was stirred for an additional 15 min at 0 °C. Ammonia solution (10 mL) was added and the reaction was stirred at room temperature overnight. The consumption of the starting amino acid was monitored by TLC. The solvent was evaporated, and the white precipitate was dissolved with EtOAc and washed with 10 % citric acid, brine, and water. The organic layer was evaporated, and product was purified by recrystallisation using EtOAc/hexane. The final product was obtained as a white solid.

N, N'-oxalyl-bis(Leu)-NH₂

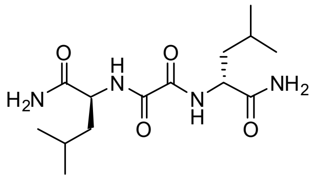

Chemical Formula: $C_{14}H_{26}N_4O_4$
Molecular Weight: 314,38

^{1}H NMR (600 MHz, DMSO) δ / ppm 8.48 (d, *J* = 8.9 Hz, 1H), 7.52 (s, 1H), 7.15 (s, 1H), 4.31 - 4.25 (m, 1H), 1.66 - 1.60 (m, 1H), 1.55 – 1.47 (m, 2H), 0.87 (dd, *J* = 10.6 Hz, 6H).

^{13}C NMR (151 MHz, DMSO) δ / ppm 173.5, 159.7, 51.9, 41.2, 24.8, 23.5, 22.0.

General scheme

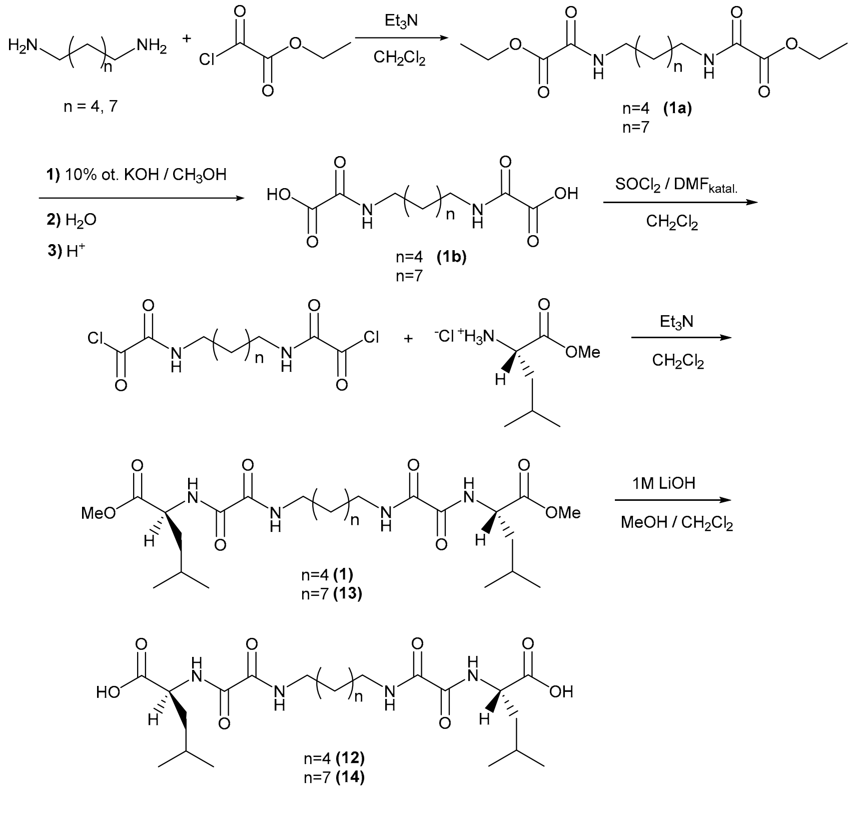

1,6-bis(ethoxyoxalamido)hexane (1a): A solution of diaminohexane (2.077 g, 17.87 mmol) in dry CH_2Cl_2 (30 mL) and TEA (4.38 ml, 39.32 mmol) were simultaneously added dropwise within 30 minutes to a cooled (-10 °C) solution of ethyl oxalylchloride (4.38 ml, 39.32 mmol) in dry CH_2Cl_2 (50 ml). The stirring was continued for 30 min at 0°C and overnight at room temperature. CH_2Cl_2 (50 ml) was added and the mixture was washed with water, 1.5% AcOH, 5% $NaHCO_3$, and again with water. The organic layer was dried (Na_2SO_4) and the solvent evaporated. Recrystallisation (CH_2Cl_2 / light petroleum) provided the title compound (3.673 g, 65 %). FTIR (KBr) $\tilde{\upsilon}$: 3322, 1747, 1733, 1681, 1544 cm^{-1}. ^1H NMR (300 MHz, $CDCl_3$, 25 °C): δ = 7.58 (bs, 2H, $CONH_{(bridge)}$), 4.20 (q, 4H, J = 7.14, $OCH_{2(ethyl)}$), 3,20 - 3,16 (m, 4H, $CH_{2(1)}$), 1,44 - 1,42 (m, 4H, $CH_{2(2)}$), 1.29-1.17 (m, 10H, $CH_{3(ethyl)}$, $CH_{2(3)}$) ppm. ^{13}C NMR (75.5 MHz, $CDCl_3$, 25 °C): δ = 160.2 (CO_{ester}); 156.5 (CONH), 62.7 ($OCH_{2(ethyl)}$), 39.2 ($CH_{2(1)}$), 28.4 ($CH_{2(2)}$), 25.8 ($CH_{2(3)}$), 13.4 ($CH_{3(ethyl)}$) ppm.

1,6-bis(hydroxyoxalamido)hexane (1b): A solution of **1a** (3.300 g, 10.43 mmol) was dissolved in 10% KOH / CH_3OH (134 mL). The reaction mixture was stirred overnight at room temperature. The methanol was evaporated, water was added, and the solution acidified with 1M HCl until pH=2 when precipitation occurred. The precipitate was filtered off, washed with water and methanol, and dried under reduced pressure. Yield: 1.813 g, 67 %. FTIR (KBr) $\tilde{\upsilon}$: 3354, 3204, 1761, 1682, 1560 cm^{-1}. ^1H NMR (300 MHz, CD_3OD, 25 °C): δ = 8.87 (m, 2H, $CONH_{bridge}$), 3.35 - 3.32 (m, 4H, $CH_{2(1)}$), 1.65 - 1.60 (m, 4H, $CH_{2(2)}$), 1.46 – 1.41 (m, 4H, $CH_{2(3)}$) ppm. ^{13}C NMR (75.5 MHz, CD_3OD, 25 °C): δ = 162.9 (COOH), 160.2 (CONH), 40.8 ($CH_{2(1)}$), 30.1 ($CH_{2(2)}$), 27.6 ($CH_{2(3)}$) ppm.

1,6-bis((O-leucylmethanol)-N-yloxalamido)hexane (1): A solution of $SOCl_2$ (5.57 mL, 76.8 mmol) and catalytic DMF (4 drops) were simultaneously added dropwise within 30 minutes to a cooled (-10 °C) solution of **1b** (1.00 g, 3.84 mmol) in dry CH_2Cl_2 (60 mL). The stirring was continued for 2 hours under reflux at 40°C. The solvent and residual HCl were evaporated under reduced pressure. This product was dissolved in dry CH_2Cl_2 (80 ml) and was simultaneously added dropwise within 30 minutes to a cooled (-10 °C) solution of H-Leu-OMe·HCl (1.536 g, 8.45 mmol) and TEA (2.25 mL, 16.14 mmol) in dry CH_2Cl_2 (40 ml). The stirring was continued overnight at room temperature. CH_2Cl_2 (50 ml) was added and the mixture was washed with water, 1.5% AcOH, 5% $NaHCO_3$, and again with water. The organic layer was dried (Na_2SO_4) and the solvent evaporated. Recrystallisation (CH_2Cl_2 / *n*-hexane) provided the title compound (1.213 g, 61 %). M. p. 180 °C. $[\alpha]_D$ = -26 (c=1 in CH_2Cl_2). ^1H NMR (300 MHz, $CDCl_3$, 25 °C): δ = 7.90 (2H, d, *J* = 9.1, NH_{Leu}), 7.64 (m, 2H, NH_{bridge}), 4.61 – 4.54 (m, 2H, $CH_{(\alpha,Leu)}$), 3.72 (s, 6H, $OCH_{3(ester)}$), 3.32 – 3.27 (m, 4H, $CH_{2(1)}$), 1.71 – 1.61 (m, 6H, $CH_{2(\beta,Leu)}$, $CH_{(\gamma,Leu)}$), 1.60 – 1.50 (m, 4H, $CH_{2(2)}$), 1.35 (m, 4H, $CH_{2(3)}$), 0.94 – 0.92 (m, 12H, $CH_{3(\delta,Leu)}$) ppm. ^{13}C NMR (75.5 MHz, $CDCl_3$, 25 °C): δ = 171.8 (COO_{ester}), 159.6 ($CONH_{Leu}$), 159.1 ($CONH_{bridge}$), 52.3 ($OCH_{3(ester)}$), 50.9 ($CH_{(\alpha,Leu)}$), 40.9 ($CH_{2(1)}$), 39.3 ($CH_{2(\beta,Leu)}$), 28.9 ($CH_{2(2)}$), 26.1 ($CH_{2(3)}$), 24.6 ($CH_{(\gamma,Leu)}$), 22.6, 21.5 ($CH_{3(\delta,Leu)}$) ppm. FTIR (KBr) $\tilde{\upsilon}$: 3295, 1741, 1656, 1524 cm^{-1}. EA calcd. for (%) $C_{24}H_{42}N_4O_8$ (517.64): C 56.01, H 8.23, N 10.89; found: C 56.25, H 7.93, N 10.86.

Determination of Gelling Properties

All gelation experiments were performed in test tubes of 12 mm in diameter.

The tested substance was placed in a test tube, and the oil was added in 500 μL portions. After each addition, the mixture was gently heated until the substance dissolved and was then allowed to cool spontaneously to

room temperature and the formation of gel was checked by test tube inversion. The procedure was repeated until the formation of a loose gel or dissolution was observed.

Gelation properties of prepared compounds were tested against various edible oils and the results are collected in Tables 1 and Table S1. The gelation efficiency of each gelator toward the specified edible oil is expressed in mL of oil that could be immobilized by 10 mg of the gelator.

All of the prepared gels are transparent and show thermoreversible gel-to-sol transitions.

In the experiments, sunflower oil (Zvijezda), soybean oil (Alfa Aeser), and olive oil (Primadonna) were used.

Determination of thixotropic property

The formed gel was subjected to external mechanical stress (shaking) until the gel is transformed into a sol state. The self-healing process (recovery to a gel state) after standing at room temperature was determined every 5 minutes by the tube inversion method and visual observation. The time of response necessary to self-heal from sol to gel state was determined at a half of the maximum gelling volume per 10 mg of the tested compound (Table S1, column recovery time (min)).

Table S1. Gelation efficiency of the compounds **4-14** toward the specified edible oil expressed as the maximal volume (V_{max}/ml) of oil that could be immobilized by 10 mg of the gelator. Thixotropic property measured at a half of the maximum gelling volume.

Compound	Sunflower oil V/ml	Recovery time*/min	Soybean oil V/ml	Recovery time/min	Olive oil V/ml	Recovery time/min
4	NG		-	-	-	-
5	3		-	-	-	-
6	24.5	no	32	no	15.2	no
7	1.8		-	-	-	-
8	1.8		-	-	-	-
9	NG		-	-	-	-
10	NG		-	-	-	-
11	2		-	-	-	-
12	NG		-	-	-	-
13	9	25 min	7	5 min	8.7	15 min
14	5**		-	-	-	-

*Self-healing properties of the gels were checked by test-tube inversion every 5 minutes. *NG= no gelation; **very weak gelation after 24 h

TEM microscopy

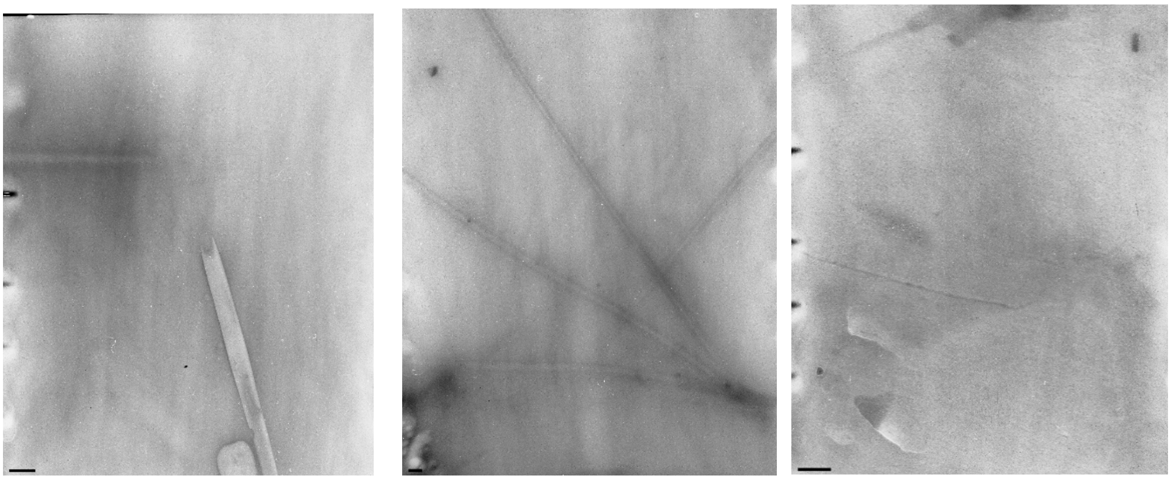

a) **b)** **c)**

Figure S1. TEM image of (a), (b) **2** sunflower oil gel (fibers and ribbons of 100-400 nm), (c) **3** sunflower oil gel (fibers and ribbons of 40 – 400 nm in diameter; bar 1 μm).

TEM images of gelator 1 in DMSO/water and tetraline

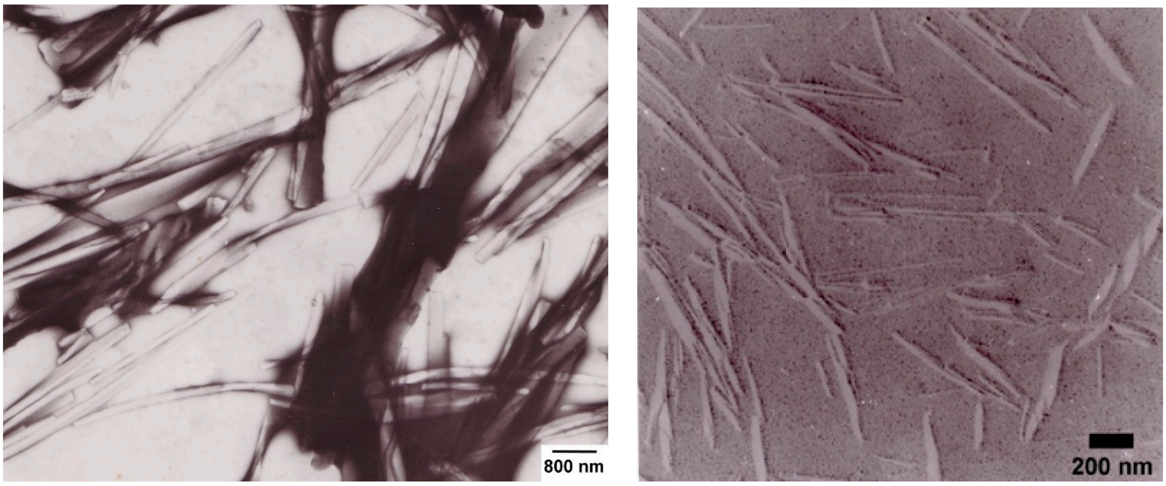

Figure S2. TEM images of a) **1** DMSO/water gel, ribbons with apparent twist (mostly 80–200 and up to 600 nm widths), b) **1** tetralin gel (Pd shadowing) fibers of 8–20 nm d values.

Gel–sol phase transitions

Table S2. Gel–sol phase transition temperatures of **1** sunflower oil gels and **1** gelled emulsions at 10% and 20% water content obtained by the dropping ball method

c / mmol L^{-1}	1 sun	1 sun/water (10%)	1 sun/water (20%)
		t / °C	
1.2	51.4	80.1	85.3
1.44	74	86.4	90.1
2	78.8	92.9	94.2
4.3	97.9	100.3	99.8
7.6	113.9	110.2	112.4
12	120.6	124.2	120.9

Oscillatory Rheology

3ITT test of 1 gels

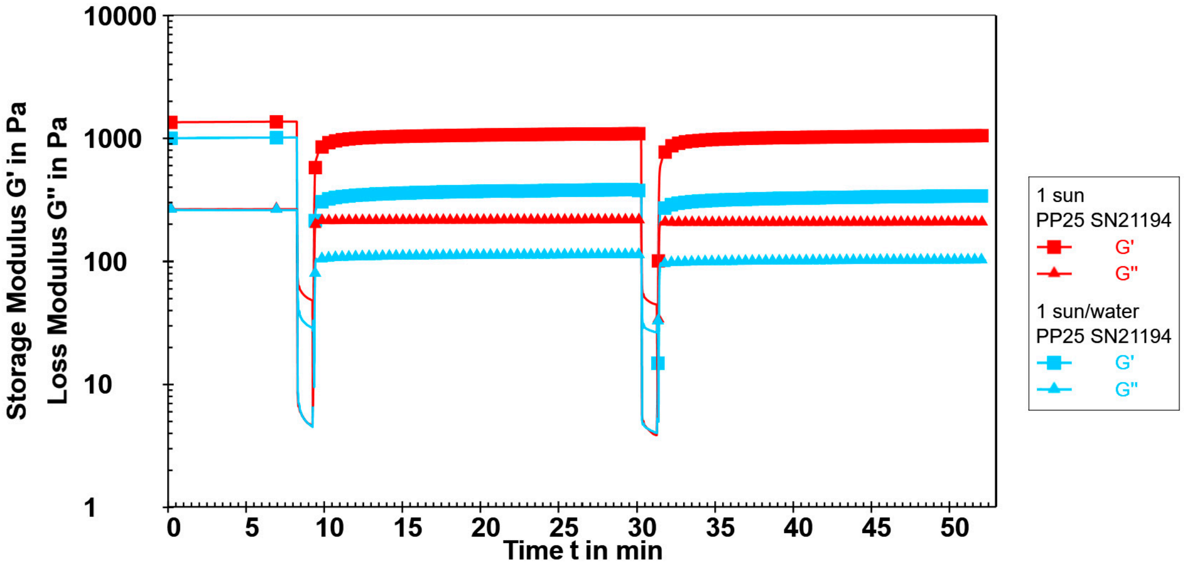

Figure S3. 3-interval thixotropy test (3ITT) (*G´* and *G´´* values) of the 0.2 wt % of **1** sunflower oil gels and **1** gelled w/o emulsion (10 % H_2O). Linear viscoelastic region (LVR): strain = 0.1%, frequency = 10 rad/s; destructive region (DR): strain = 100%, frequency = 10 rad/s; recovery region: linear viscoelastic region (LVR): strain = 0.1%, frequency = 10 rad/s.

Table S3. Self-healing properties of 0.2 wt % of **1**, **2,** and **3** sunflower oil gels and **1** gelled w/o emulsion (10 % H_2O, **1** sun/water) determined in 2nd consecutive recovery cycle of 3-interval thixotropy test in the 3.-5. interval.

	3.-5. interval		
**Sample**	**Recovery (*t* = 60 s)/%**	**Recovery (*t* = 300 s)/%**	**Recovery (*t* = 900 s)/%**
1 sun	80.1	91.0	95.0
3 sun	62.1	93.6	99.5
1 sun/water	76.8	84.1	88.6
2 sun	69.5	84.3	91.9

Controlled release properties of oil gels

To investigate a release profile of the selected analytes from prepared gels, the simple LC–MS method for determination of *acetylsalicylic acid, ibuprofen,* and hydrocortisone was developed.

Chemicals

Acetylsalicylic acid for standard preparation was purchased from TCI (Zwijndrecht, Belgium) with the purity > 98,0%. Ibuprofen and hydrocortisone as the pharmaceutical reference materials were obtained from Merck.

Ethanol absolute was purchased from Riedel-de Haën (*Seelze*, Germany). MiliQ® water (18.2 $M\Omega cm^{-1}$, purified by MiliQ water purification system (Millipore, Bedford, MA, USA)) and HPLC gradient-grade methanol (J.T.Baker, Center Valley, USA) were used with analytical-grade formic acid (FA) (Acros Organics, Geel, Belgium) for mobile phase preparation.

Preparation of standard solutions

Stock solutions of *acetylsalicylic acid, ibuprofen,* and hydrocortisone were prepared as 1 mg/mL solutions in methanol.

Sample preparation and LC–MS condition

Release of acetylsalicylic acid

The gels with approximately 2 mg acetylsalicylic acid were prepared in a well-closed glass vial and stored at room temperature. The gel surface was overlaid with 2 ml of MiliQ water. At every selected time point after the addition of water on the gel surface, including immediately after addition, two 50 μL sample aliquots were sampled for determination of the release rate of acetylsalicylic acid from prepared gels. The sampled aliquots were replaced with an equal volume of fresh MiliQ water to maintain the total volume of water over the gel. In one aliquot, a corresponding volume of stock solution was added (spiked sample); in the second,

the same volume of methanol was added (non-spiked sample). An amount of 5 μL of clear solution was injected on the LC column. During analysis, all instrumental blank samples were negative.

Quantification of the analyte was performed by monitoring the difference in the peak areas of the acetylsalicylic acid in the non-spiked sample and the spiked sample prepared with the addition of the known concentration of the standard.
LC–MS analysis was carried out using an Agilent Technologies 1200 series HPLC system equipped with a binary pump, a vacuum membrane degasser, an automated autosampler, and injector interfaced with 6420 triple quadropole mass spectrometer with electrospray ionization source (ESI) (Agilent Technologies Inc., Palo Alto, CA, USA).
The analysis was performed on Zorbax XDP C18 column (75 x 4.6 mm, 3.5 μm particle size) (Agilent Technologies Inc., Palo Alto, CA, USA). Solvents for the analysis were 0.1% formic acid (FA) in water (solvent A) and methanol (solvent B). The gradient was applied as follows: 0 min 70% A, 0-10 min 70% A-0% A, 10-12 min 0% A, 12.1-15 min 70% A. Flow rate was 0.5 mL/min.

The electrospray ionization source was operated in a positive mode and samples were detected in the single ion monitoring (SIM) mode. *Acetylsalicylic acid was monitored at m/z 203 as a* $[M+Na]^+$ adduct. Fragmentor voltage for *acetylsalicylic acid* was set at 100 V.
The desolvation gas temperature was 300 ⁰C with a gas flow rate of 8.0 L/min. The capillary voltage was 4.0 kV.
The retention time of *acetylsalicylic acid was 7,4 min.*

All data acquisition and processing were performed using Agilent MassHunter software.

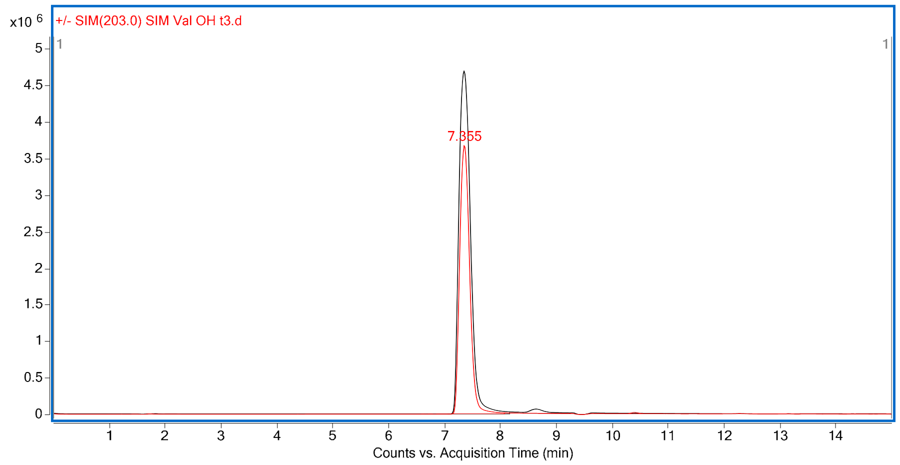

Overlaid SIM chromatograms of non-spiked (red) and spiked samplse (black) obtained by releasing *acetylsalicylic acid* incorporated in gel Val-OH at t1

Release of ibuprofen

The sample prep procedure was the same as for acetylsalicylic acid, except that the gel was overlaid with 2 mL of ethanol instead of water due to better solubility of ibuprofen in ethanol. Ibuprofen was monitored at *m/z 229 as a* $[M+Na]^+$ adduct in a positive ionization SIM mode. Other chromatographic and ionization parameters were the same. The retention time of ibuprofen was 12,3 min.

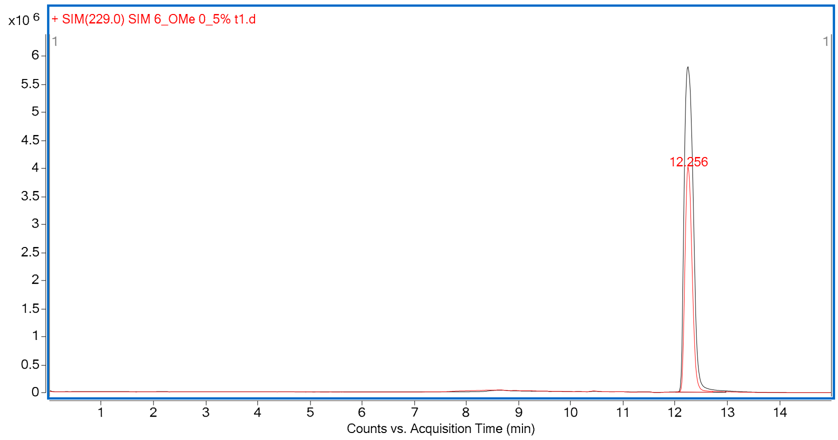

Overlaid SIM chromatograms of non-spiked (red) and spiked samples (black) obtained by releasing ibuprofen incorporated in gel 6_OMe_0,5% at t1

Release of hydrocortisone

The gel was overlaid with 2 mL of ethanol due to better solubility of hydrocortisone in ethanol. Hydrocortisone was monitored at *m/z 363 as* $[M+H]^+$ ion in a positive ionization SIM mode. Fragmentor voltage was set at 135 V. Other ionization parameters were the same. The gradient eluating was 0 min 70% A, 0-12 min 70% A-0% A, 12-15 min 0% A, 15.1-20 min 70% A. Other chromatographic parameters were the same. The retention time of hydrocortisone was 10,5 min.

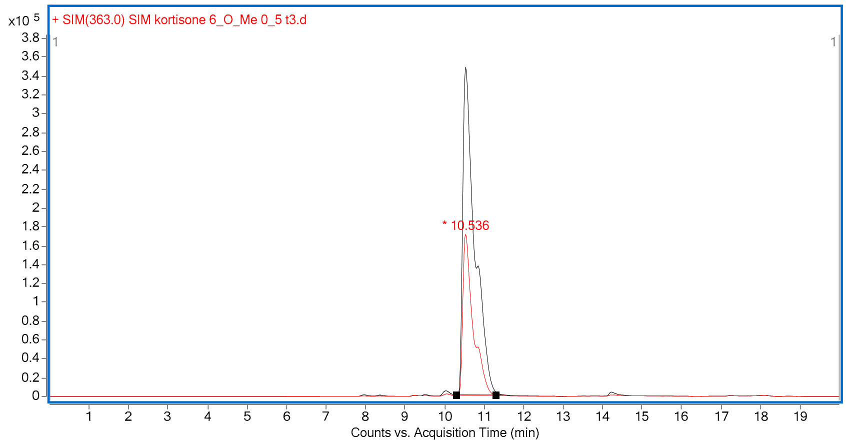

Overlaid SIM chromatograms of non-spiked (red) and spiked samples (black) obtained by releasing hydrocortisone incorporated in gel 6-OMe_0,5% at t3

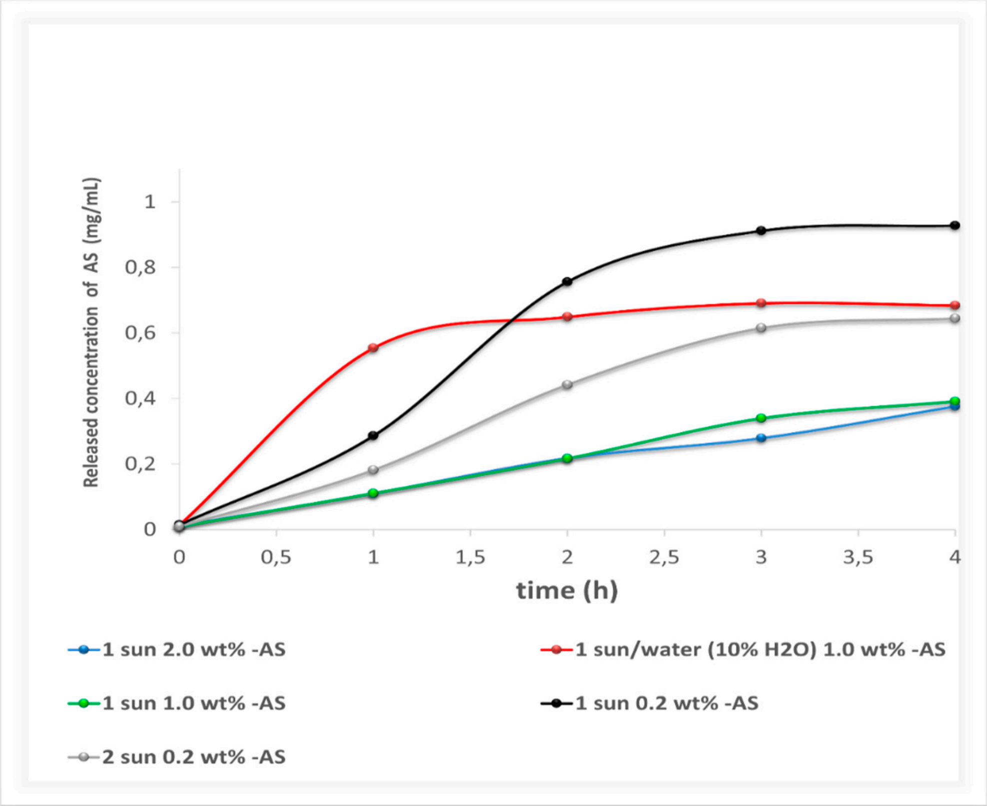

Figure S4. Controlled release profile of acetylsalicylic acid from **1** sunflower oil gels at different gelator concentrations (0.2 wt% black; 1 wt% green; 2 wt% blue); **1** gelled w/o emulsion (10% H_2O; 1 wt% red) and **2** sunflower oil gel (0.2 wt% grey) from initial point to until 4h at 25 °C.

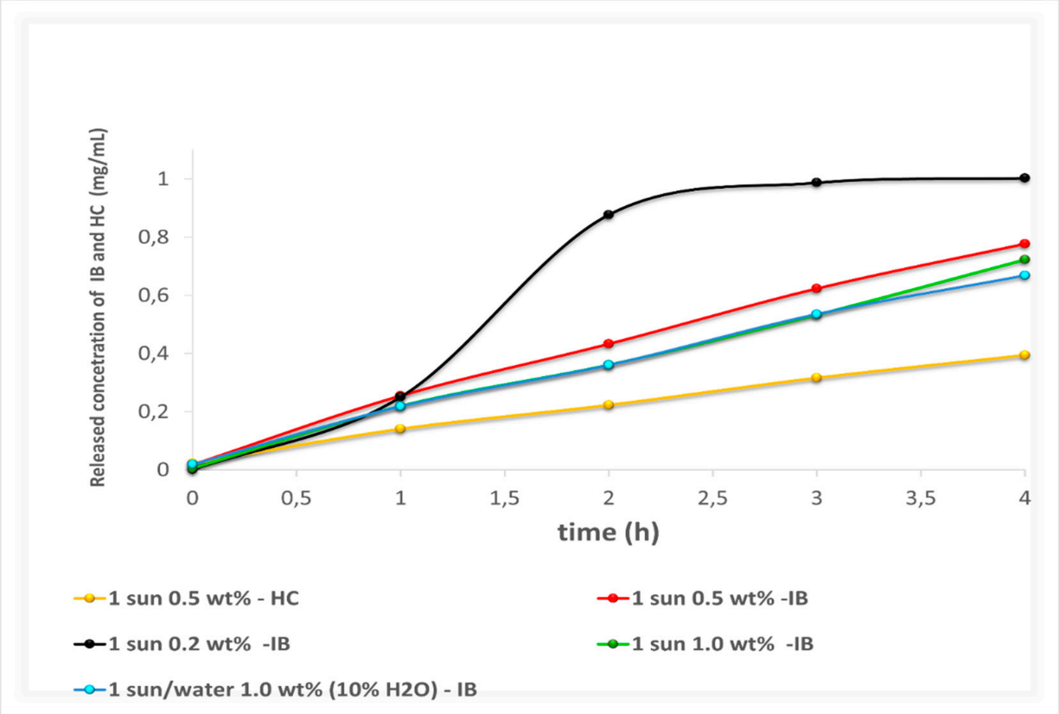

Figure S5. Controlled release profile of ibuprofen from **1** sunflower oil gels at different gelator concentrations (0.2 wt% black; 0.5 wt% red; 1 wt% green); **1** gelled w/o emulsion (10% H_2O; 1 wt% blue); and sustained release of hydrocortisone of **1** sunflower oil gel (0.5 wt% yellow) from initial point to until 4h at 25 °C.

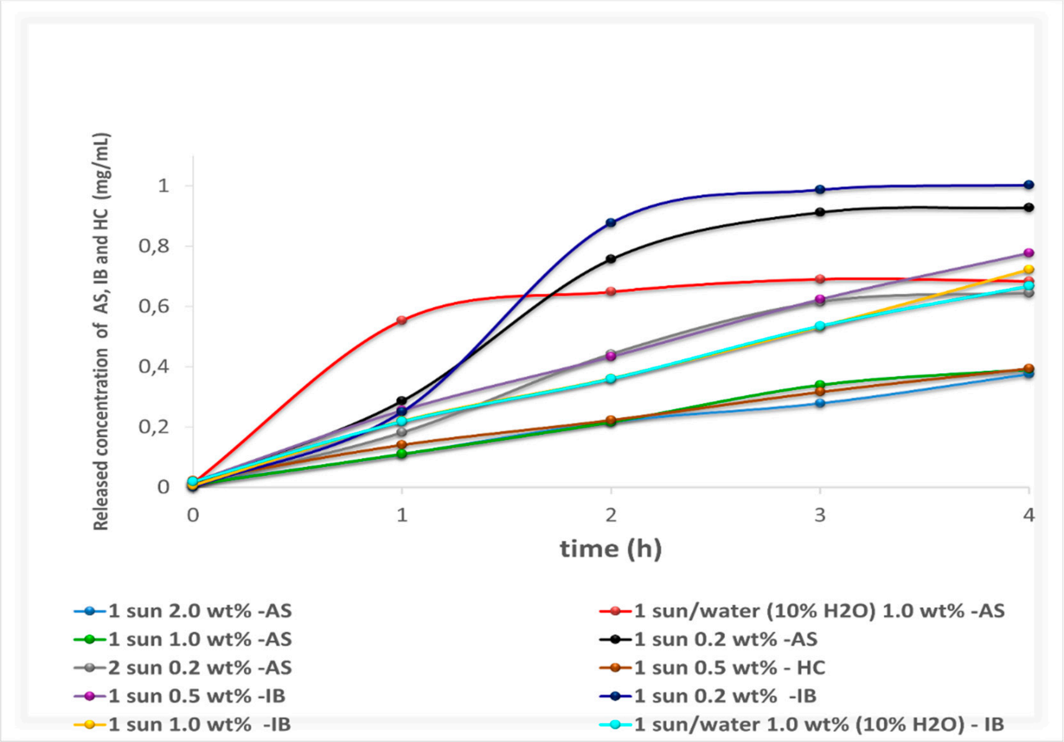

Figure S6. Controlled release profile of acetylsalicylic acid from **1** sunflower oil gels at different gelator concentrations (0.2 wt% black; 1 wt% green; 2 wt% blue); **1** gelled w/o emulsion (10% H_2O; 1 wt% red); **2** sunflower oil gel (0.2 wt% grey); sustained release profile of ibuprofen from **1** sunflower oil gels at different gelator concentrations (0.2 wt% blue; 0.5 wt% violet; 1 wt% yellow); **1** gelled w/o emulsion (10% H_2O; 1 wt% cyan); and sustained release of hydrocortisone of **1** sunflower oil gel (0.5 wt% brown) from initial point to until 4h at 25 °C.

Fat substitute experiments in food application

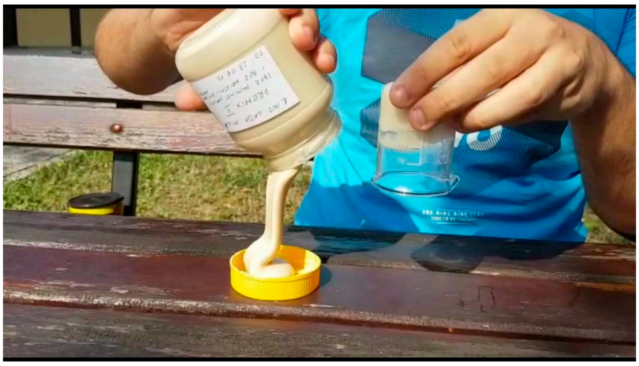

a)

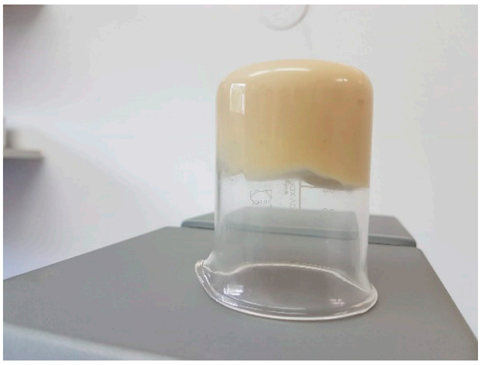

b)

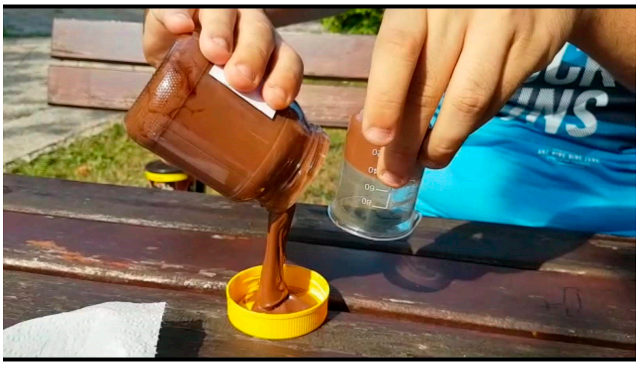

c)

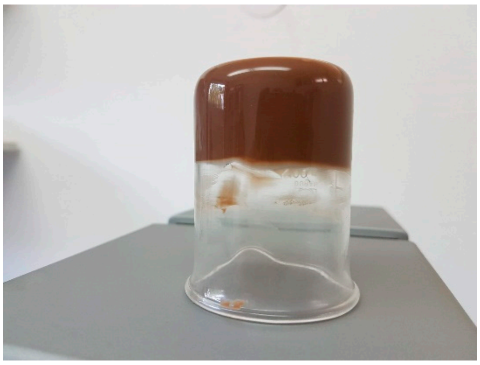

d)

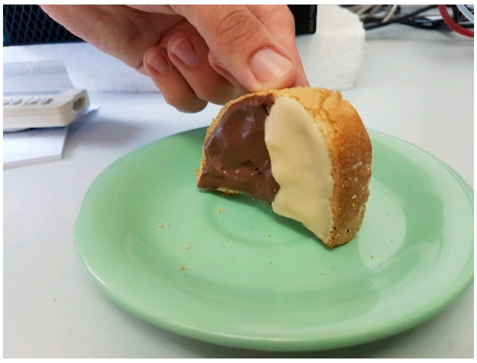

e)

Figure S7. The comparison of a) gelled milk spread (0.1 wt % of gelator **1** and sunflower oil) and milk spread with sunflower oil, without palm fats (flowing), b) gelled milk spread, c) gelled cocoa spread (0.2 wt % of gelator **1** and sunflower oil) and cocoa spread with sunflower oil, without palm fats (flowing), d) gelled cocoa spread, e) application of gelled cocoa and milk spread on bread.

Stability of 1 gelled w/o emulsions in sunflower oil

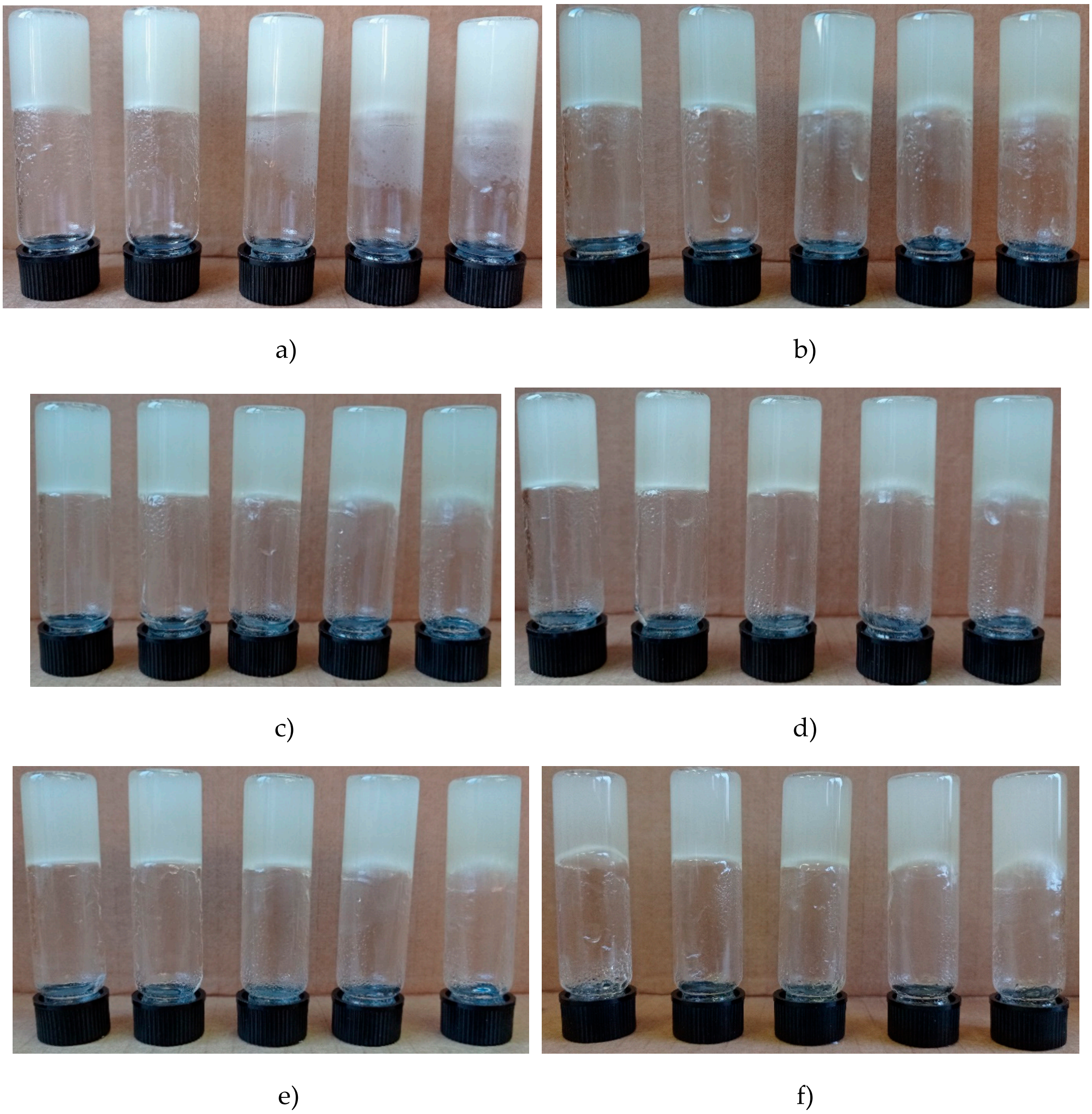

Figure S8. Stability of **1** gelled w/o emulsions in sunflower oil (10 % H_2O *v/v*) for concentrations of 1.44 mmol/L; 2.0 mmol/L; 4.3 mmol/L; 7.6 mmol/L; and 12.0 mmol/L in ascending order from the left to the right; a) after 1 day, b) 1 week, c) 2 weeks, d) 3 weeks, e) 1 month, and f) 2 months.
